# A novel approach to enhance methane production in anaerobic digestion of waste activated sludge via pre-enrichment by a microbial consortium of degrading fungi

**DOI:** 10.1128/aem.00008-26

**Published:** 2026-02-27

**Authors:** Kun Dai, Xiao-Mei Zhu, Yan-Lin Hu, Yu-Er Cao, Xing-Chen Huang, Xiao-Fei Yang, Raymond Jianxiong Zeng, Fang Zhang

**Affiliations:** 1 Center of Wastewater Resource Recovery, College of Resources and Environment, Fujian Agriculture and Forestry University 602381, Fuzhou, Fujian, China; Shanghai Jiao Tong University, Shanghai, China

**Keywords:** native fungi, polysaccharides in cell wall, waste-activated sludge, microbial consortium of degrading fungi, chitinase

## Abstract

Both fungi and bacteria contribute to the efficiency of activated sludge processes. However, fungal degradability has historically been overlooked in the anaerobic fermentation of waste-activated sludge (WAS). This study investigated the role of an enriched microbial consortium of degrading fungi (MCDF) in WAS fermentation. MCDF was initially enriched using a mixture of WAS and two mesophilic anaerobic sludges with chitin as the substrate. Fungal diversity and key cell wall polysaccharides, including chitin, mannan, and glucans, were identified in WAS. The addition of enriched MCDF to WAS significantly increased methane production by 35% and disrupted the floc structure through degradation of native fungi. The enriched MCDF also utilized fungal polysaccharides from four fungal species (*Candida albicans*, *Trichosporon asahii*, *Geotrichum sp.,* and *Magnusiomyces capitatus*) and facilitated the release of intracellular organics. *Proteinophilum* and *Petrimonas* were identified as the main producers of chitinase (EC 3.2.1.14). Additional microbial producers were identified for mannanase (EC 3.2.1.78), β−1,4-glucanase (EC 3.2.1.4), and β−1,3-glucanase (EC 3.2.1.6). The relative activities of these four hydrolases in WAS were only 0.01%–12.3% of those in enriched MCDF. These results highlight the multifunctional potential of MCDF enrichment in WAS digestion, including degradation of fungal cell wall polysaccharides, release of intracellular fungal organics, and hydrolysis of bacterial extracellular organics. Overall, this study demonstrates that the destruction of native fungi in WAS provides a promising approach to enhance methane production and anaerobic digestion efficiency in wastewater treatment plants.

**IMPORTANCE:**

Both fungi and bacteria contribute to maintaining the floc structure of waste-activated sludge (WAS). This study highlights that MCDF enrichment significantly enhances methane production and disrupts the floc structure through the degradation of native fungi in WAS. Fungal diversity and key fungal cell wall polysaccharides—chitin, mannan, and glycans—were identified in WAS. The MCDF consortium was shown to utilize extracellular organics from four fungal species (*Candida albicans*, *Trichosporon asahii*, *Geotrichum sp*., and *Magnusiomyces capitatus*) and promote the release of intracellular organics. Multi-omics analysis revealed *Proteinophilum* and *Petrimonas* as the main chitinase producers in MCDF. Additional hydrolytic activities were observed: *Fermentimonas* and *Petrimonas* for mannanase, *Mesotoga* and *Proteinophilum* for β−1,4-glucanase, and *Syntrophorhabdus* for β−1,3-glucanase. Notably, the activities of these four hydrolases were significantly higher in MCDF than in WAS, indicating that MCDF is the main source of key functional enzymes. These findings highlight the multifunctional potential of MCDF enrichment in enhancing methane production during WAS digestion.

## INTRODUCTION

The effectiveness of activated sludge processes in wastewater treatment plants (WWTPs) largely depends on the activities of various bacteria, eukaryotes (fungi, algae, and protozoa), and archaea (1–4). For example, More et al. reported the presence of filamentous fungi such as *Penicillium*, *Aspergillus*, and *Trichoderma* in sewage sludge (5). Wei et al. reported that the fungus-to-bacterium ratio ranged from 0.08% to 1.15% in the collected 30 activated sludge samples (6). A higher fungus-to-bacterium ratio of 5% was deemed as a key factor to assure the aggregation of sludge floc and reactor health (7). Notably, a fungus affiliated with *Candidatus Alysiosphaera* was identified as a key connector in the microbial network, highlighting synergistic interactions mediated by carbon metabolism and nitrogen cycling (8). Moreover, WWTPs generate large amounts of waste-activated sludge (WAS) (9). Anaerobic digestion is a widely used biotechnology for recovering CH_4_ or volatile fatty acids (VFAs) from WAS (10–12). However, the efficiency of this biotechnology is limited by the poor hydrolysis of macromolecular organic matter within extracellular polymeric substances (EPS) secreted by both bacteria and fungi.

To address this limitation, thermal and chemical pretreatment technologies have been developed to disrupt the structural integrity of WAS flocs and enhance the hydrolysis of encapsulated organics (13–15). For example, thermal pretreatment at 200°C for 60 min reduced the median particle size (D50) of WAS from 105.3 μm to 61.1 μm and increased the soluble chemical oxygen demand (SCOD) from 0.6 g/L to 3.8 g/L (16). However, pretreatment technologies are constrained by high operating costs and the potential release of poorly degradable organic compounds (14, 17). Therefore, the enrichment of an alginate-degrading consortium may enhance EPS hydrolysis and increase CH_4_ production from WAS (18, 19), highlighting the advantages of microbial-based approaches for WAS fermentation.

Several studies have shown that fungi are widely distributed in WWTPs (3, 4, 20). For example, phylogenetic analyses revealed that native fungal communities in three WWTPs included members from 6 phyla and 361 genera (3). The most prevalent fungal phylotypes, mainly from the classes *Saccharomycetes* and *Chytridiomycetes*, play key roles in degrading organic matter and trace organic contaminants, such as dyes and endocrine disruptors (20). In addition to bacterial EPS, native fungi contribute to maintaining floc structure (21–23). Fungal pellets can form well-settling aggregates via self-immobilization (21). Filamentous fungi reinforce flocs, enabling the formation of larger and stronger aggregates (22, 23). Geng et al. recently reported that fungal pellets, with their large specific surface area and abundant functional groups, can serve as a framework to strengthen granule structure (24). However, microbial strategies for disintegrating the WAS structure through the degradation of native fungi remain limited.

The fungal cell wall is crucial for cell viability. Therefore, the degradation of the fungal cell wall is a key step for disrupting structural integrity and enhancing methane production during WAS digestion. The main polysaccharides in fungal cell walls include chitin (a polymer of N-acetyl-D-glucosamine, GlcNAc, with β-glucosidic bonds), β-glucans (glucose polymers with 1,3- and 1,4-β-glucosidic bonds), and mannan (a polymer of mannose with α-glucosidic bonds) (25, 26). Chitin, which accounts for 10%–20% of cell wall polysaccharides, exhibits high tensile strength and maintains overall cell wall integrity (25, 27). Notably, chitin is the most recalcitrant polysaccharide in the fungal cell wall under anaerobic conditions (28, 29). For example, the anaerobic degradation of 2 g/L of chitin by enriched mesophilic anode anaerobes required over 45 days (29). These findings suggest that chitin could serve as a new target for enhancing WAS digestion through the enrichment of a microbial consortium capable of degrading fungi.

Intracellular organics, which account for 10%–70% of total cellular organics (27, 30), can be released and utilized upon cell wall disruption. Particularly, lysozyme is a commonly used enzymatic method to release intracellular organics in bacteria and enhance CH_4_ production from WAS (31). Hydrolases such as chitinase (EC 3.2.1.14), mannanase (EC 3.2.1.78), β−1,4-glucanase (EC 3.2.1.4), and β−1,3-glucanase (EC 3.2.1.6) are involved in the hydrolysis of fungal polysaccharides (25). Therefore, enriching a fungus-degrading consortium (MCDF) with chitin as the key substrate can degrade cell wall polysaccharides, release intracellular organics, and promote CH_4_ production from WAS.

This study examined the presence of fungi and their secreted polysaccharides in WAS. Methane recovery from native fungi and their polysaccharides (chitin, glucan, and mannan) was evaluated using the enriched MCDF. Key bacteria and extracellular enzymes were identified through a combination of high-throughput sequencing, metagenomics, and metaproteomics. Moreover, the mechanism of native fungus degradation during WAS digestion was elucidated. These findings provide valuable insights into the role of fungal polysaccharides in WAS digestion and support the development of novel microbial strategies to enhance methane recovery.

## RESULTS

### Characterization of fungi and their polysaccharides in a local WWTP

**Fig 1:**
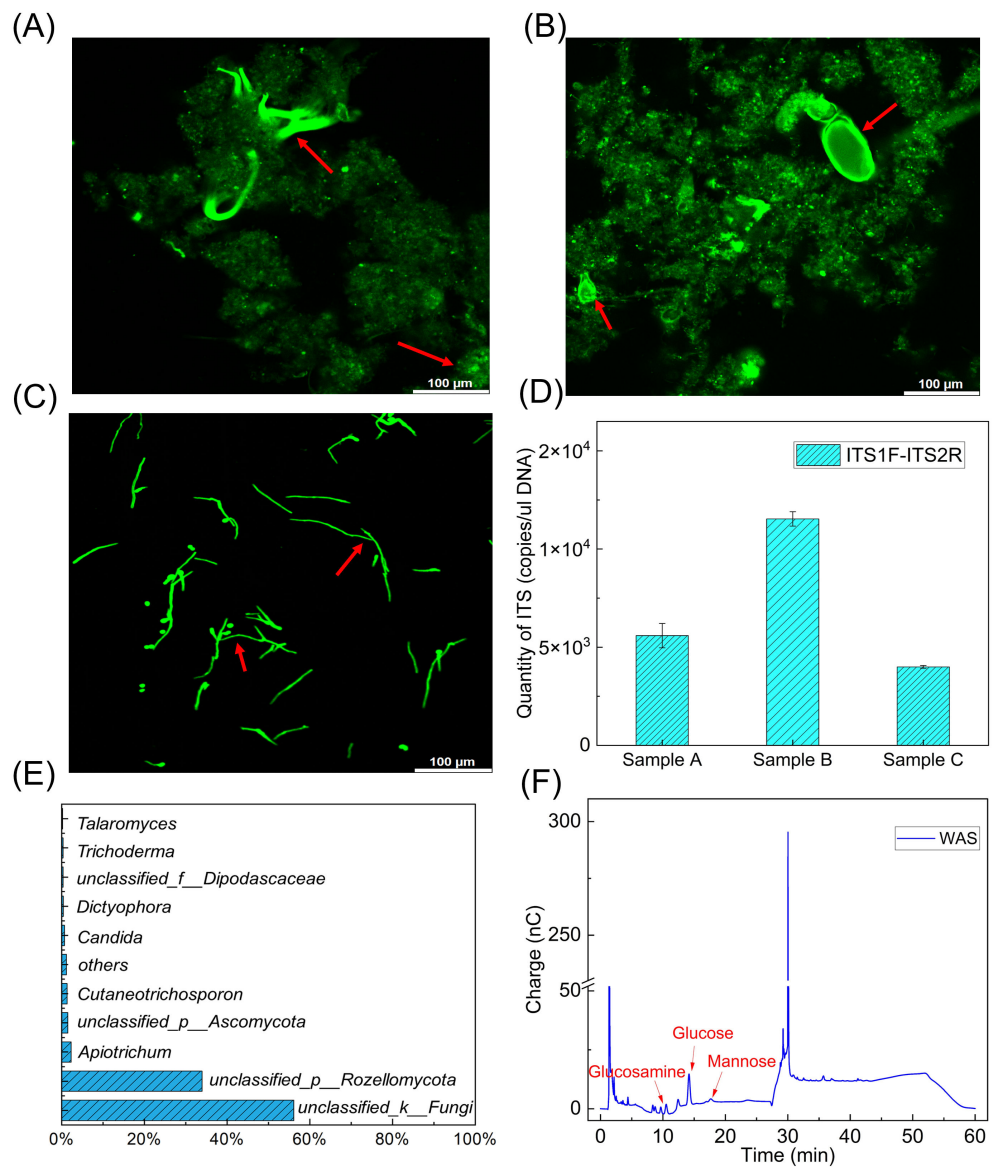
Occurrences of fungi and polysaccharides in WAS. Calcofluor White staining of (**A** and **B**) WAS and (**C**) a typical fungus of *Trichosporon asahii*; (**D**) quantitative PCR (qPCR) of ITS1F/ITS2R for fungal samples collected in the bioreactor (sample A), influent of the settler (sample B), and WAS (sample C); (**E**) fungal diversity at the level of genus in WAS; (**F**) identified monomers of fungal polysaccharides in WAS.

Both filamentous and branched structures with green fluorescence were observed in WAS using Calcofluor White staining (Fig. 1A and B; Fig. S1). These structures were consistent with the morphological characteristics of fungi, such as filamentous fungi and *Trichosporon asahii* (Fig. 1C). Fungi were detected throughout the main technological stages in a local WWTP, as indicated by fungal copy numbers obtained using the universal ITS1F/ITS2R primers (Fig. 1D). The number of fungal genes in samples A and B was 0.56 ± 0.06 × 10^4^ copies/μL DNA and 1.15 ± 0.004 × 10^4^ copies/μL DNA, respectively. In the collected WAS, this number decreased to 0.4 ± 0.0007 × 10^4^ copies/μL DNA. *Rozellomycota* was the predominant fungal genus in WAS (Fig. 1E; Fig. S2; Table S2). However, 56.1% of the fungi remained unidentified. The monomers of these polysaccharides, including mannose, glucose, and glucosamine, were detected in the EPS hydrolysates (Fig. 1F). These results highlight the role of fungi as structural components in WAS, contributing to floc stability. The degradation of these organics may disrupt the floc structure and enhance methane production, as investigated in the following sections.

### CH_4_ production from native fungi and floc structure changes by MCDF

**Fig 2:**
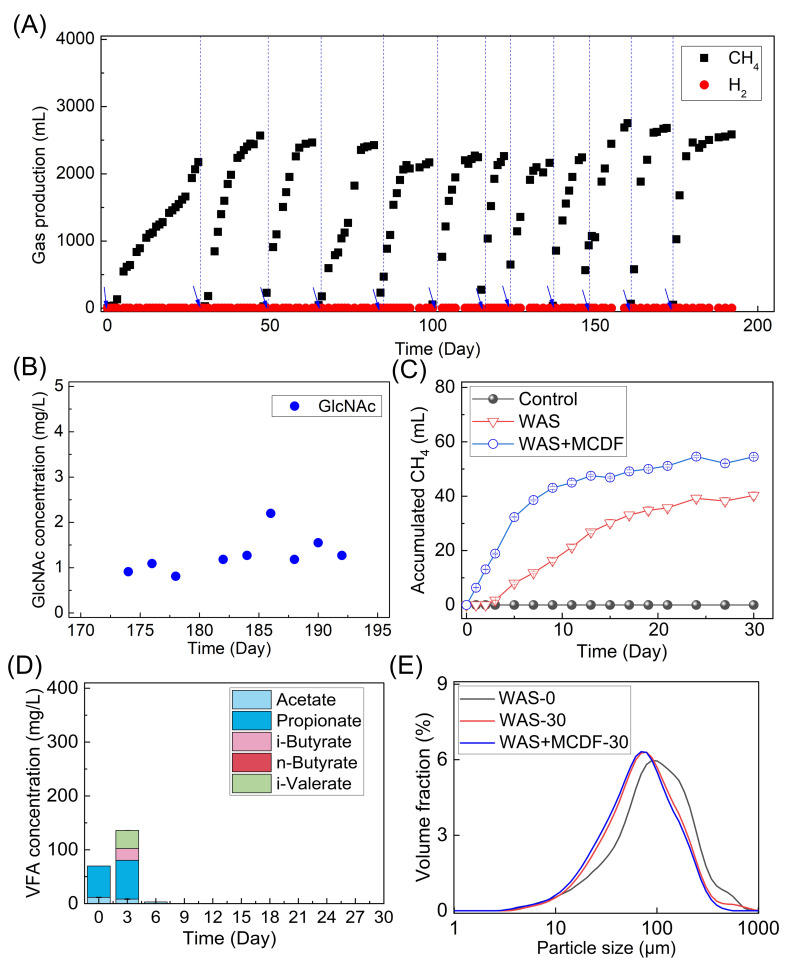
Promoting WAS digestion via degrading native fungi by the enriched MCDF. (**A**) Methane production during the MCDF enrichment, (**B**) the release of GlcNAc (N-acetyl-D-glucosamine, the monomer of chitin) in the last cycle (days 174–192), (**C**) methane production in the groups of Control (no WAS), WAS (no MCDF), and WAS+MCDF, (**D**) VFA accumulation in the WAS+MCDF group, and (**E**) change in particle size.

A 2-L mesophilic reactor was operated with 5 g/L of chitin to enrich MCDF over 200 days. Methane production during the last 4 cycles reached around 2,700 mL (equivalent to 270 mL/g chitin, Fig. 2A). Accumulated VFAs (Fig. S3), including acetate, propionate, and butyrate, remained below 0.1 g/L. H_2_ levels were consistently below 0.1%, and the final biomass concentration reached ∼1.8 g/L. Considering both chitin degradation and methane production, the chemical oxygen demand (COD) balance exceeded 92%. The chitin monomer (i.e., GlcNAc) was below 2 mg/L during the last cycle (days 174–192, Fig. 2B). The produced N-NH_4_^+^ concentration increased from 350 mg/L to 600 mg/L (Fig. S3), supporting the effective degradation of chitin by the enriched MCDF. Three biomass samples were collected on days 60, 120, and 180 to examine the dynamic bacterial diversity at the initial (cycle 3), intermediate (cycle 7), and final (cycle 12) stages, respectively.

Methane production in the WAS group alone was 40.26 ± 1.35 mL (equivalent to 88.29 mL/gVSS, Fig. 2C). The addition of enriched MCDF to WAS increased methane production in the MCDF+WAS group to 54.51 ± 0.31 mL (119.5 mL/gVSS), representing a 35% increase. No methane was detected in the control group without WAS. Enriched MCDF also promoted the conversion of accumulated VFAs, with only minor amounts of propionate and i-valerate detected in the WAS+MCDF group (Fig. 2D). In the WAS group, ~300 mg/L of VFAs was detected and consumed after 15 days. By the end of the experiment in the WAS+MCDF group (Fig. S3), the number of fungal genes detected via qPCR reached 0. This indicates that native fungi in the collected WAS were degraded by enriched MCDF. Dosing MCDF disrupted the floc structure, leading to a lower particle size (D90) of 166.9 μm (Fig. 2E; Table S3). These results confirm that enriched MCDF can effectively disintegrate WAS flocs and enhance methane production. Therefore, the conversion of both typical fungi and their cell wall polysaccharides was investigated in the following sections.

### Methane production from four typical fungi using the enriched MCDF

**Fig 3:**
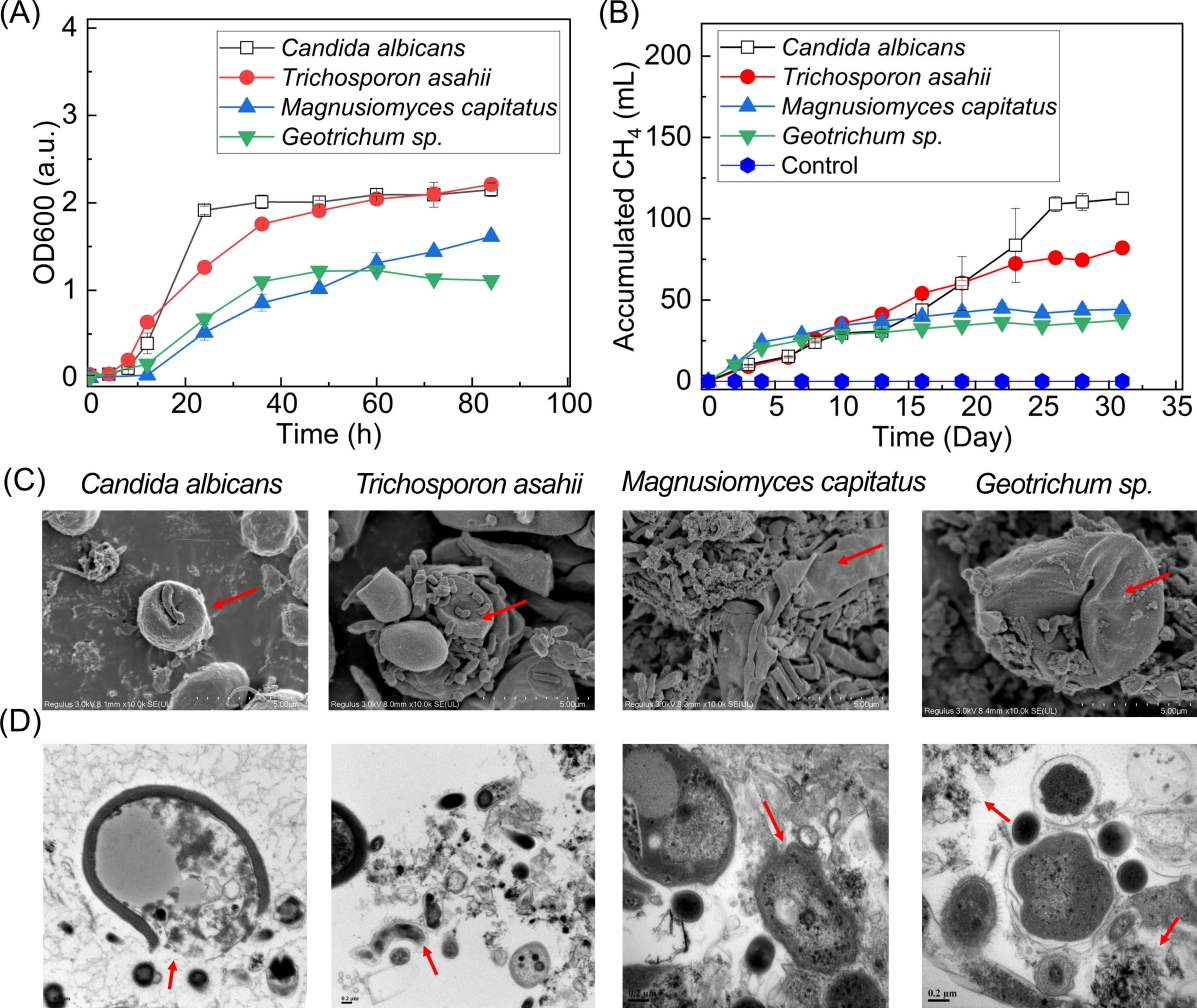
Degradation of four typical fungi by the enriched MCDF. (**A**) Growth curve, (**B**) methane production from four fungi and control (no fungi added), (**C**) scanning electron microscopy (SEM), and (**D**) transmission electron microscopy (TEM) pictures of four typical fungi, from left to right: *Candida albicans*, *Trichosporon asahii*, *Magnusiomyces capitatus*, and *Geotrichum sp*. The arrows point to the pictures of fungi.

Four fungi—*Candida albicans*, *Trichosporon asahii*, *Geotrichum sp*., and *Magnusiomyces capitatus—*were selected based on their relative abundances in WAS: *Candida* (0.7%), *Trichosporon* (0.2%), *Geotrichum* (0.02%), and *Magnusiomyces* (<0.001%) (Fig. 1E). Figure 3A shows the growth curves of the four typical fungi. After 84 h, the OD_600_ values of *Candida albicans*, *Trichosporon asahii*, *Magnusiomyces capitatus*, and *Geotrichum sp*. were 2.2 ± 0.08, 2.0 ± 0.03, 1.6 ± 0.03, and 1.5 ± 0.02, respectively. The collected fungi were refreshed in the inorganic medium to remove residual organics and then used as substrates for the enriched MCDF. Methane production from *Candida albicans*, *Trichosporon asahii*, *Magnusiomyces capitatus*, and *Geotrichum sp*. was 112.5 ± 3.5 mL, 82.0 ± 2.0 mL, 44.4 ± 2.8 mL, and 37.6 ± 0.1 mL, respectively (Fig. 3B). VFAs, including acetate and propionate, were detected in all 4 groups (Fig. S4). These VFAs were completely consumed to produce methane within 30 days through syntrophic oxidation of acetate and propionate, acetoclastic methanogenesis, and hydrogenotrophic methanogenesis (11, 12). No H_2_ was detected during methane production. Overall, the methane yields corresponded to the OD values of the four fungi.

Figure 3C (SEM) and 3D (TEM) show the morphology of the four fungi after anaerobic degradation by MCDF. SEM images revealed that MCDF tightly adhered to the fungal surface, and fungal cells exhibited withered, wrinkled, or locally lysed morphologies. TEM images further indicated that the structural integrity of fungal cell walls and membranes was disrupted, leading to the release of insoluble intracellular compounds, such as organelle fragments. MCDF bacteria directly colonized the fungal cell wall, suggesting that they may accelerate the disintegration of fungal residues. Therefore, enriched MCDF could potentially damage fungi and release intracellular organics, thereby enhancing methane production in WAS (Fig. 2C). The enzyme activities and conversion of fungal cell wall polysaccharides are discussed in the following section.

### Conversion of fungal cell wall polysaccharides and enzyme activities by enriched MCDF

**Fig 4:**
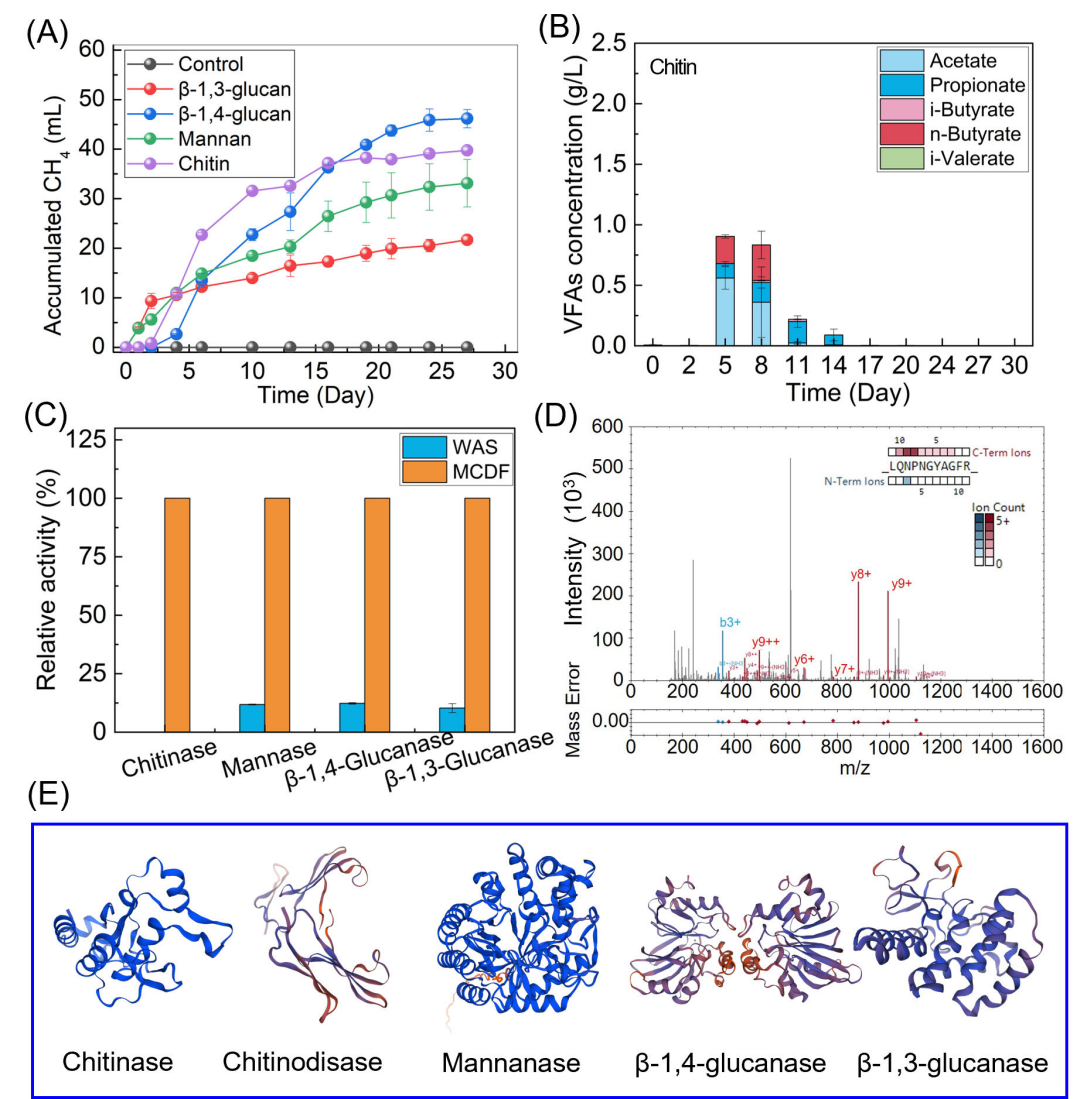
Degradation of four typical polysaccharides in the fungal cell wall by the enriched MCDF and enzymatic activities. (**A**) Methane production, (**B**) VFA accumulation from chitin, (**C**) relative activities of chitinase, mannanase, β−1,4-glucanase, and β−1,3-glucanase in MCDF and WAS, (**D**) MS2 spectrum of identified peptide in chitinase, (**E**) and 3D structure of five hydrolases identified in MCDF.

Chitin, mannan, β−1,4-glucan, and β−1,3-glucan are typical fungal cell wall polysaccharides and were used as substrates for the enriched MCDF. Methane production from chitin, mannan, β−1,3-glucan, and β−1,4-glucan was 40.0 ± 0.7 mL (133.3 mL/g), 33.2 ± 1.4 mL (110.7 mL/g), 21.7 ± 0.8 mL (72.3 mL/g), and 46.2 ± 1.8 mL (154.0 mL/g), respectively (Fig. 4A). No methane was detected in the control group without substrates. VFAs, including acetate and propionate, were detected as intermediates and completely consumed by the end of digestion (Fig. 4B and S5). Similar to Fig. 2B, the concentrations of monomers GlcNAc, mannose, and glucose remained below 2 mg/L throughout the process. Hydrolysis by extracellular enzymes is a key step in organic conversion. Only minor chitinase (EC 3.2.1.14) activity was detected in WAS, with relative activity below 0.01% of that in enriched MCDF (Fig. 4C). The relative activities of mannanase (EC 3.2.1.78), β−1,4-glucanase (EC 3.2.1.4), and β−1,3-glucanase (EC 3.2.1.6) were 11.8%, 12.3%, and 10.3% of the MCDF levels, respectively. These results confirm that enzyme activities excreted by MCDF were significantly higher than those in WAS, thereby enhancing the utilization of fungal polysaccharides. However, these enzymatic assays shall be further determined by using purified enzymes for typical polysaccharides to support the positive role of enriched MCDF.

The extracellular enzymes involved in MCDF-mediated hydrolysis were further identified via metaproteomic analysis. Multiple protein bands were detected by SDS-PAGE, with molecular weights ranging from 10 kDa to 180 kDa (Fig. S6). Five MCDF enzymes—MCDF_k97_71210_1_1 (chitinase, EC 3.2.1.14), MCDF_k97_18863_1_1 (chitinodisase, EC 3.2.1.52), MCDF_k97_112905_37_1 (mannanase, EC 3.2.1.78), MCDF_k97_67082_40_1 (β−1,3-glucanase, EC 3.2.1.6), and MCDF_k97_51468_1_1 for β−1,4-glucanase (EC 3.2.1.4)—were identified with sequence similarities of 56.8%–100% and low e-values from 0 to 3 × 10⁻³⁶ (Fig. 4D; Fig. S6; Table S4). The 3D structures of these enzymes were constructed using SWISS-MODEL (a fully automated protein structure homology-modeling server) based on sequences identified by metaproteomics (Fig. 4E). These results indicate that enriched MCDF can degrade typical fungal organic matter. Chitin, as a structural skeleton, provides mechanical strength to fungi. Chitinase secretion decomposes chitin, thereby disrupting the fungal structure. This process releases intracellular compounds and promotes the utilization of fungal organic matter, consistent with the observations in Fig. 2 and 3.

### Bacterial diversity and metabolic mechanisms of fungal polysaccharides in enriched MCDF

**Fig 5:**
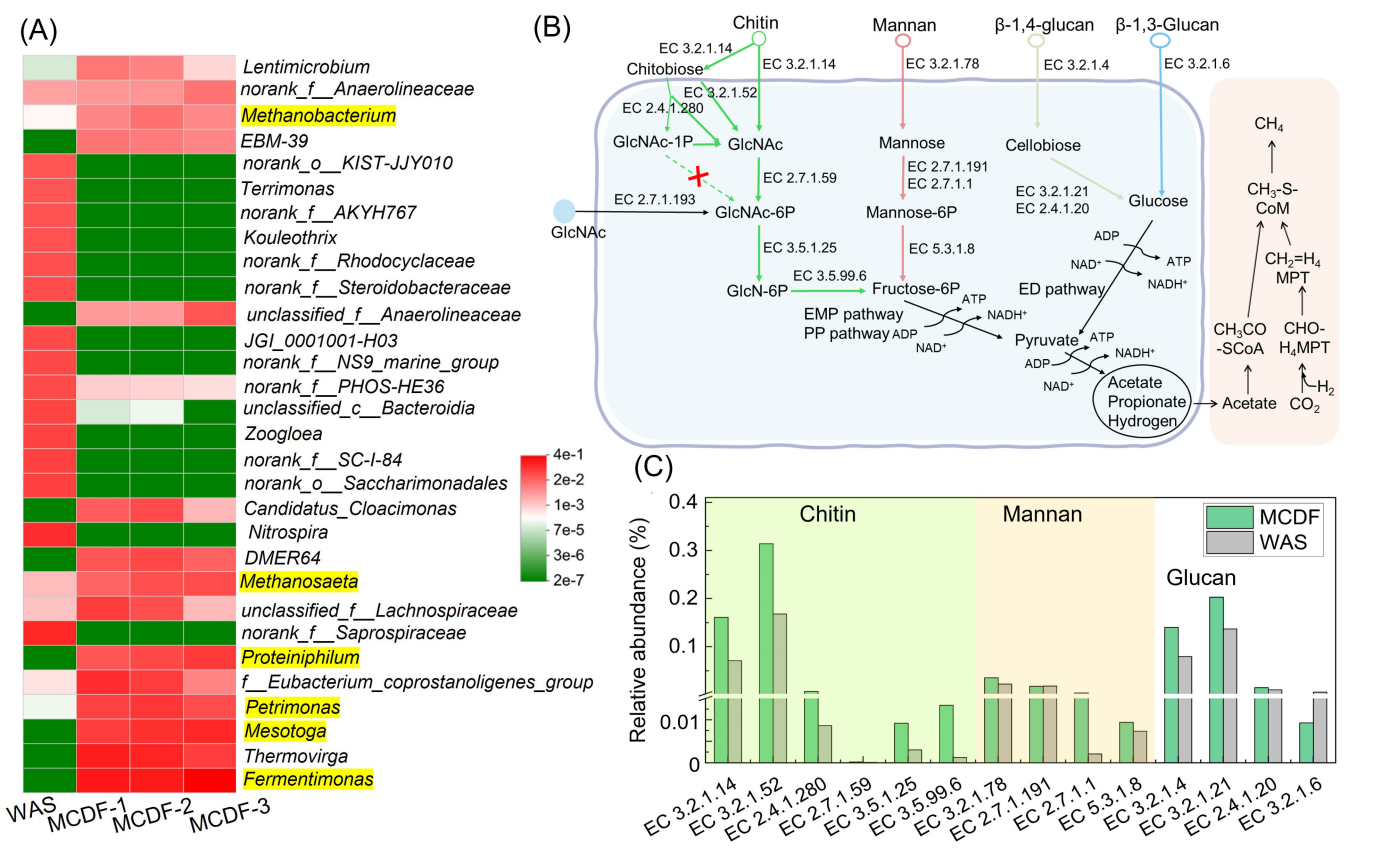
Microbial diversity and metabolic pathways of enriched MCDF. (**A**) Microbial relative abundance at the genus level; (**B**) enriching the metabolic pathways of MCDF; (**C**) relative abundance of key genes.

The bacterial diversity of enriched MCDF was characterized by high-throughput sequencing. The Shannon curve and Sob index (Table S5; Fig. S7) indicated that the sequencing depth was sufficient to capture the microbial diversity. Figure 5A summarizes the microbial diversity of the top 30 genera, with dominant genera including *Fermentimonas*, *Mesotoga*, *Proteinophilum*, and *Thermovirga*. A total of 3 genera—including *Fermentinomas* (increased from 0.001% in WAS to 42.0% in MCDF-3), *Proteinophilum* (from 0.002% in WAS to 8.2% in MCDF-3), and *Thermovirga* (from 0.002% in WAS to 7.4% in MCDF-3)—were mainly involved in the catabolism of proteins and amino acids (32–34). *Mesotoga* (whose abundance increased from 0.001% in WAS to 13.8% in MCDF-3) is a member of the Thermotogae class and can hydrolyze carbohydrates under anaerobic conditions (35). *Methanobacterium* converts H_2_ and CO_2_ to methane, and *Methanosaeta* converts acetate to methane and CO_2_ (36). These two methanogens were also identified in MCDF-3 with the percentages of 0.8% and 5.0%, respectively. Additionally, VFAs (main propionate) are converted to hydrogen (or formate) and acetate by syntrophs of *Syntrophobacter*, *Syntrophorhabdus*, and *Syntrophomonas* (37, 38). The relative abundances of these bacteria were below 0.1%.

The metabolic pathways by which enriched MCDF utilizes fungal organic matter were further investigated via metagenomic analysis (Fig. 5B; Fig. S8 to S12). Chitin is hydrolyzed into GlcNAc and chitosan by chitinase EC 3.2.1.14, with chitosan further converted to GlcNAc by EC 2.4.1.280 and EC 3.2.1.52. Mannan is hydrolyzed into mannose by mannanase (EC 3.2.1.78). β−1,4-glucan is hydrolyzed into glucose by EC 3.2.1.4, EC 3.2.1.21, and EC 2.4.1.20. β−1,3-glucan is hydrolyzed into glucose by EC 3.2.1.6. These monosaccharides are then converted into fructose-6-phosphate and metabolized to pyruvate via the Embden-Meyerhof-Parnas (EMP) and pentose phosphate (PP) pathways. The hydrolysates are transformed into acetate, propionate, butyrate, and methane.

The relative abundances of bacteria involved in degrading fungal cell wall polysaccharides were higher in MCDF than in WAS (Fig. 5C; Tables S6 to S10). Notably, *Proteinophilum* and *Petrimonas* (4.8% in MCDF-3) were the main producers of chitinase (EC 3.2.1.14). *Fermentimonas* and *Petrimonas* mainly produced mannanase (EC 3.2.1.78). *Mesotoga* and *Proteinophilum* were the main genera secreting β−1,4-glucanase (EC 3.2.1.4). *Syntrophorhabdus* (0.13% in MCDF-3) was the main producer of β−1,3-glucanase (EC 3.2.1.6). These findings indicate that enriched MCDF can effectively utilize fungal cell wall polysaccharides, consistent with the results shown in Fig. 2C, 3B and 4A. Moreover, these results highlight the advantage of using a mixed culture for the degradation of fungal organics during WAS digestion.

## DISCUSSION

### Native fungus degradation: a multifunctional strategy for enhancing WAS digestion

The activated sludge process, a widely used biological treatment in WWTPs, inevitably produces large amounts of WAS (39). WAS digestion is mainly limited by the hydrolysis of macromolecular EPS by multiple hydrolases, which restricts subsequent acidogenesis and methanogenesis (40–42). In EPS, uronic acids from alginate and polygalacturonate can bind multivalent cations, forming a gel-like WAS structure (43–45). Recently, enrichment of a *Bacteroides*-dominant consortium can increase methane production from WAS by 115% through improved alginate hydrolysis (19). Chitin, mannan, and glucan are the main polysaccharides in fungal cell walls (25). This study presents a microbial strategy to disrupt fungal structures through the secretion of multiple hydrolases capable of hydrolyzing cell wall polysaccharides, including chitin, mannan, 1,3-glucan, and 1,4-glucan. Notably, chitinase activity (EC 3.2.1.14) in WAS was only 0.01% of that observed in the enriched MCDF (Fig. 4A). In contrast, the relative activities of mannanase (EC 3.2.1.78), β−1,4-glucanase (EC 3.2.1.4), and β−1,3-glucanase (EC 3.2.1.6) ranged from 10.3% to 12.3% of MCDF levels. Combining MCDF with alginate-degrading bacteria as a microbial cocktail may further enhance WAS digestion, which will be investigated in future studies.

In addition to uronic acids, the hydrolyzed gelling EPS contains monomers such as neutral sugars (glucose and mannose) and amino sugars (galactosamine and glucosamine) (9, 43). For example, glycosaminoglycans—composed of hexosamine, galactose, and uronic acid—are key structural components of aerobic granular EPS (46). Peptidoglycans, which constitute over 70% of the bacterial cell wall, consist of amino sugars, cell wall acids, and side-chain amino acids. Recently, Wang et al. reported that degrading amino sugars can enhance methane production during WAS digestion (36). This study further revealed that dosing MCDF disrupts fungal structures and releases intracellular organics (Fig. 3), making it challenging to distinguish between bacterial and fungal polysaccharides. Therefore, the enhanced methane production observed with the enriched MCDF can be attributed to the combined utilization of fungal cell walls and other degradable bacterial organics in WAS.

### Environmental implications of fungal polysaccharides in floc formation

The activated sludge process, used for over a century, remains the core technology for wastewater treatment in WWTPs (44). To elucidate the roles of EPS and cations in floc formation, several theories have been formulated, including the alginate theory, Derjaguin–Landau–Verwey–Overbeek (DLVO) theory, and divalent cation bridging (DCB) theory (22, 47, 48). According to the alginate and DCB theories, uronic acids from alginate and polygalacturonate can bind multivalent cations such as Ca^2+^, forming a stable gelling structure in the outer layer of WAS flocs (9, 43, 44). For example, galacturonate, accounting for 7.2% (wt/wt), was detected in hydrolysates of gelling EPS (43). Wang et al. further revealed that insoluble and hydrophobic EPS (including both polysaccharides and proteins) can be recovered using a cation exchange resin combined with ultrasonication (49). Pronk et al. reported that after extracting uronic acids from WAS, a large amount of insoluble and hydrophobic EPS could still be recovered using the heat-acetic acid method (50). Consequently, research on insoluble and hydrophobic organics within the DLVO framework remains limited.

Ryan et al. reviewed the microbial synthesis of exopolysaccharides in lactic acid bacteria and found that heteropolysaccharides contain 3 to 8 repeating units of several monosaccharides, including glucose and GlcNAc (51). Similarly, An et al. identified 2 amino sugars (GlcNAc and N-acetylfucosamine) in *Zoogloea bacterium* (52). The degradation of amino sugars, such as chitin, is considered particularly challenging under anaerobic conditions (28, 53). For example, Li et al. reported that enriched mesophilic anode anaerobes required over 45 days to degrade 2 g/L of chitin (29). These results indicate that chitin is resistant to degradation by native WAS bacteria, which maintain the WAS structure according to the DLVO theory. Chitin, mannan, and β-glucan in fungal cell walls are typical insoluble polysaccharides (25). Therefore, this study provides new insights into WAS floc formation. However, the detailed, stratified distribution of these fungal polysaccharides in WAS remains to be elucidated in future research.

### MCDF-based bioaugmentation strategy proposed for anaerobic digestion of WAS in WWTPs

The use of a composite substrate containing fungal and bacterial exopolysaccharides can enrich a more robust microbial consortium capable of secreting diverse extracellular hydrolases with high activity. Because EPS is the main organic product of genera such as *Zoolgoea* and *Nitrospira* in activated sludge (54), its degradation by MCDF could further support this approach in future studies. These functions can be investigated through a combination of digestor operation, enzymatic activity assays, and multi-omics analyses (55). Additionally, over 200 million tons of mushroom cultivation waste are produced each year globally, with the chitin content reaching up to 10% of dry weight (56). These mushroom wastes can serve as a low-cost substrate for MCDF enrichment.

Thus, an MCDF bioaugmentation reactor would be constructed to enhance methane production from the co-digestion of WAS and mushroom wastes. To maintain the high hydrolase activity of chitinase and mannanase in the MCDF, a sidestream bioreactor fed with concentrated WAS and the mushroom wastes shall also be constructed before seeding the MCDF into the main large-scale anaerobic digester periodically. A comparative economic analysis of MCDF-based bioaugmentation and other pretreatment methods, such as thermal processing, will also be conducted to promote the application of this bioaugmentation strategy. Overall, this study provides a potential multifunctional strategy via MCDF enrichment. This approach enables the degradation of polysaccharides from both fungal cell walls and bacterial EPS and the release of intracellular organics from fungi.

In summary, the enriched MCDF can degrade both pure fungi (*Candida albicans*, *Trichosporon asahii*, *Geotrichum sp.,* and *Magnusiomyces capitatus*) and their polysaccharides, such as chitin, mannan, and β-glucan. *Proteinophilum* and *Petrimonas* were identified as the main producers of chitinase. Other contributors included *Fermentimonas* and *Petrimonas* for mannanase, *Mesotoga* and *Proteinophilum* for β−1,4-glucanase, and *Syntrophorhabdus* for β−1,3-glucanase. In WAS, the relative activities of these four hydrolases were only 0.01%–12.3% of those in the enriched MCDF. These results highlight the multifunctional potential of MCDF in WAS digestion, including floc disruption and a 35% increase in methane production.

## MATERIALS AND METHODS

### Inoculum preparation and activation of a fungus-degrading microbial consortium

The WAS sample was obtained from the concentrated pond of a local WWTP in Jinshan (Fuzhou, China), which operates a sequencing batch reactor with a capacity of 50,000 m^3^/d. Inocula for MCDF enrichment were collected from a mixture of WAS and two mesophilic anaerobic sludges (14, 36) at a randomly decided volatile suspended solid (VSS) ratio of 4:1:1. Key WAS parameters, including pH, SCOD, total chemical oxygen demand (TCOD), VSS, and SS, are summarized in Table S1. The inocula, with an initial VSS of 2.5 g/L, were sparged with N_2_ (>99.99%) for 15 min and added to a mesophilic (35°C) batch reactor. The anaerobic reactor had a total volume of 3 L and a working volume of 2 L. Chitin (5 g/L) was added 12 times on days 0, 29, 48, 64, 83, 100, 115, 124, 137, 147, 161, and 174 in a fed-batch mode to enrich MCDF. The inorganic medium, containing 50 mM PBS buffer, was prepared as described previously (11). The pH was manually maintained at 7.0 ± 0.2 via dosing with 2 M NaOH or HCl. The temperature was maintained at 35°C using a water bath. On days 83 and 147, half of the reactor liquid (~1 L) was replaced with fresh medium. The concentrations of released NH_4_^+^, GlcNAc (chitin monomer), CH_4_, H_2_, and VFAs (including acetate, propionate, i-butyrate, n-butyrate, and i-valerate) were measured.

### Enriched MCDF-mediated WAS digestion

To investigate the effect of enriched MCDF on methane production from WAS, three experimental groups were established (*n* = 3 each): control, WAS+MCDF, and WAS alone. The MCDF effluent was centrifuged at 8,000 rpm for 10 min to remove the supernatant and resuspended in an equal volume of inorganic medium. In the WAS+MCDF group, 20 mL of MCDF was mixed with 40 mL of WAS at a ratio of 0.08 gVSS/gVSS and placed in 120 mL serum bottles. The bottles were sparged with N_2_ (> 99.99%) for 15 min. In the WAS group, 40 mL of WAS was combined with 20 mL of inorganic medium in 120 mL serum bottles to assess methane production from WAS alone. In the control group, 20 mL of MCDF was mixed with 40 mL of inorganic medium in 120 mL serum bottles to evaluate methane production from MCDF alone. All groups were incubated at 35°C on a shaker at 120 rpm. Methane and VFA production were monitored, while changes in WAS particle size were analyzed.

### Cultivation and degradation of four fungal species by the enriched MCDF

Four fungal species—*Trichosporon asahii*, *Candida albicans*, *Magnusinomyces capitatus*, and *Geotrichum sp*.—were purchased from Beina Biotechnology. The fungi were cultured in the YM medium containing yeast extract (3 g/L), glucose (10 g/L), and peptone (5 g/L). Cultures were incubated in a mesophilic shaking chamber at 28°C and 120 rpm. Fungal growth was monitored using OD_600_ measurements.

To evaluate fungal conversion by the enriched MCDF, *Trichosporon asahii*, *Candida albicans*, *Magnusinomyces capitatus*, and *Geotrichum sp*. were harvested and centrifuged at 8,000 rpm for 15 min to remove the culture medium. The fungal biomass was then resuspended in the inorganic medium described in Section 2.1. For each species, 40 mL of the fungal suspension was mixed with 20 mL of MCDF and transferred into 120 mL serum bottles (*n* = 3). The bottles were sparged with N_2_ (>99.99%) for 15 min. A control group containing only 20 mL of MCDF and 40 mL of inorganic medium was prepared (*n* = 3) to assess methane production from residual organics in the MCDF solution. All bottles were incubated in the batch mode at 35°C in a shaking chamber operated at 120 rpm. The concentrations of methane, H_2_, and VFAs, including acetate, propionate, i-butyrate, n-butyrate, and i-valerate, were quantified.

### Degradation of fungal cell wall polysaccharides and hydrolase activity

Four cell wall polysaccharides—chitin, mannan, β−1,4-glucan, and β−1,3-glucan—were used as substrates to evaluate the conversion of fungal organic matter by the enriched MCDF. Five experimental groups were established (*n* = 3 each): control (no substrate), chitin, mannan, β−1,4-glucan, and β−1,3-glucan. For the substrate treatments, 5 g/L of polysaccharides, 20 mL of enriched MCDF, and 40 mL of inorganic medium were added to 120 mL serum bottles. The bottles were sparged with N_2_ (>99.99%) for 15 min. In the control group, no substrate was added (*n* = 3). Only 20 mL of enriched MCDF and 40 mL of inorganic medium were included. The produced methane, H_2,_ and VFAs of acetate, propionate, i-butyrate, n-butyrate, and i-valerate were all determined.

The enzyme solution was collected from the culture supernatant of the MCDF reactor. The supernatant was centrifuged at 8,000 rpm for 10 min and filtered through a 0.45 μm microporous membrane to remove residual microorganisms. Enzyme solutions from WAS were also obtained from the corresponding supernatants. Chitinase (EC 3.2.1.14), mannanase (EC 3.2.1.78), β−1,4-glucanase (EC 3.2.1.4), and β−1,3-glucanase (EC 3.2.1.6) activities were determined using commercial assay kits (Solarbio, Wuhan, China). Relative enzyme activities were calculated using MCDF values as the reference.

### Chemical analysis and statistical methods

The activities of chitinase (EC 3.2.1.14), mannanase (EC 3.2.1.78), β−1,4-glucanase (EC 3.2.1.4), and β−1,3-glucanase (EC 3.2.1.6) were measured using commercial assay kits (Solarbio, Wuhan, CN). GlcNAc, glucose, and mannose concentrations were determined using the DNS method at 540 nm. NH_4_^+^ concentrations were measured through the salicylate method. Ethanol and VFAs in the liquid phase were quantified via gas chromatography (7890, Agilent, United States). CH_4_ and H_2_ concentrations in the headspace were analyzed using a separate gas chromatograph (SP7890, Lunan, China). Changes in WAS particle size were assessed using a particle size analyzer (Nanosizer ZS instrument, Malvern Co., UK).

All experiments were performed in triplicate. Statistical significance was assessed using analysis of variance followed by a least significant difference test. Differences were considered statistically significant at *P* < 0.05.

### Fungal distribution and diversity via qPCR and high-throughput sequencing

The morphologies of fungi in WAS and *Trichosporon asahii* were visualized using Calcofluor White staining (57). After anaerobic digestion by the enriched MCDF, residues of *Trichosporon asahii*, *Candida albicans*, *Magnusinomyces capitatus*, and *Geotrichum sp*. were collected via centrifugation at 8,000 rpm for 10 min to remove the supernatant. The morphologies of these residues were analyzed via SEM (Hitachi Regulus 8100, Japan) and TEM (Hitachi H-7650, Japan).

Four samples were collected from the WWTP process: the bioreactor (sample A), influent of the settler (sample B), collected WAS (sample C), and sludge after WAS digestion by the enriched MCDF (sample D). DNA was extracted from each sample. The universal ITS1F/ITS2R primer, targeting the fungal ITS1 region, was used for the analyses of real-time quantitative PCR and fungal diversity in WAS. Four collected samples were performed to quantify fungi using the Real-Time PCR System (ABI7300, USA). Then, the fungal diversity in WAS (sample C) was analyzed on a MiSeq PE 300 sequencer. Raw reads were processed on the Majorbio Cloud platform (58).

### Diversity, hydrolases, and metabolic pathways of the enriched MCDF

DNA was extracted from four samples —WAS and enriched MCDF collected on days 60, 120, and 180—designated WAS, MCDF-1, MCDF-2, and MCDF-3, respectively. Bacterial community sequencing was performed using primers 341F-806R on an Illumina MiSeq PE 300 platform. Raw sequencing reads were processed on the Majorbio Cloud platform (58).

Extracellular enzymes in the enriched MCDF on day 180 were analyzed via metaproteomics. Details are provided in the Supporting Information. Proteins were collected from the MCDF supernatant and analyzed using a Vanquish Neo UHPLC system coupled with an Orbitrap Astral mass spectrometer (Thermo Fisher, USA). Raw data were processed and searched against the UniProt and NCBI databases. The 3D structures of the identified hydrolases in MCDF were modeled using SWISS-MODEL (59).

The metabolic pathways involved in fungal polysaccharide degradation in enriched MCDF-3 were identified via metagenomic analysis using the NovaSeq 6000 platform (Majorbio, China). Details are provided in the Supporting Information. Metabolic pathways and their encoded enzymes were annotated based on KEGG using the Majorbio Cloud platform (58).

## Data Availability

The sequencing data of fungal diversity in WAS have been deposited in the NCBI database under BioProject accession PRJNA1359761. The 16S rRNA gene sequencing data for samples WAS, MCDF-1, MCDF-2, and MCDF-3 have been deposited in the NCBI database under BioProject accession PRJNA1359746. The metaproteomic data of MCDF have been deposited in the ProteomeXchange Consortium via the iProX partner repository under data set ID PXD071625. The metagenomic sequencing data have been deposited in the NCBI database under BioProject accession PRJNA1369787.

## SUPPLEMENTAL MATERIAL

Supplemental material aem.00008-26-s0001.docx Tables S1 to S10; Fig. S1 to S12.

ASM does not own the copyrights to Supplemental Material that may be linked to, or accessed through, an article. The authors have granted ASM a non-exclusive, world-wide license to publish the Supplemental Material files. Please contact the corresponding author directly for reuse.
