# Supplementary material for: A novel approach to enhance methane production in anaerobic digestion of waste activated sludge via pre-enrichment by a microbial consortium of degrading fungi
Source: Appl Environ Microbiol. 2026 Feb 27;92(3):e00008-26. doi: 10.1128/aem.00008-26 (PMC12997835; doi:10.1128/aem.00008-26)
Supplement: Supplemental material — Tables S1 to S10; Fig. S1 to S12. [file aem.00008-26-s0001.docx]

**Supporting Information**

**A novel approach to enhance methane production in anaerobic digestion of waste activated sludge via pre-enrichment by a microbial consortium of degrading fungi**

Kun Dai ^a^, Xiao-Mei Zhu ^a^, Yan-Lin Hu ^a^, Yu-Er Cao ^a^, Xing-Chen Huang ^a^, Xiao-Fei Yang ^a^, Raymond Jianxiong Zeng ^a^, Fang Zhang ^a, *^

^a^ Center of Wastewater Resource Recovery, College of Resources and Environment, Fujian Agriculture and Forestry University, Fuzhou, Fujian 350002, China

* Correspondence concerning this article should be addressed to Fang Zhang at [zhfang@mail.ustc.edu.cn](mailto:zhfang@mail.ustc.edu.cn), Tel/Fax: +86 591 83303682.

SI has 23 pages in total, including 10 tables and 12 figures.

**S1 Parameters of waste activated sludge and detailed information for metagenomic and metaproteomic analyses**

The WAS sample was collected from the Jinshan wastewater treatment plant (Fuzhou City, China). The characteristics, including pH, total COD (TCOD), soluble COD (SCOD), suspended solids (SS), and volatile suspended solids (VSS) followed the Standard Methods for the Examination of Water and Wastewater (1).

Table S1 Parameters of waste activated sludge and extracted EPS

| Parameters | Waste activated sludge |
| --- | --- |
| pH value | 7.3 ± 0.1 |
| SS (g/L) | 21.6 ± 1.1 |
| VSS (g/L) | 11.4 ± 0.2 |
| TCOD (g/L) | 19.5 ± 1.5 |
| SCOD (g/L) | 0.09 ± 0.01 |
| Soluble protein (mg/L) | 54.5 ± 1.1 |
| Soluble polysaccharides (mg/L) | 3.9 ± 0.5 |
| Soluble humic acid (mg/L) | 52.6 ± 0.4 |

**Detailed information for metagenomic and metaproteomic analyses**

Extracellular enzymes in the enriched MCDF were finally analyzed by metaproteomic analysis. The extracellular enzymes were collected from the supernatant of MCDF. The collected protein was digested with Trypsin and dried by vacuum concentrator. The peptides were analyzed by a Vanquish Neo UHPLC system coupled to an Orbitrap Astral mass spectrometer (Thermo Fisher, USA) using the uPAC High Throughput column (75 μm×5.5 cm, Thermo, USA) at Majorbio (Shanghai, China). These data were acquired using an Orbitrap Astral mass spectrometer operated in the Data-independent acquisition (DIA) mode. Raw DIA data were processed by the Spectronaut software (Version 19) with a standard protocol in Majorbio Cloud^2^ and compared against the UniProt, NCBI, and KEGG databases. The detailed parameters are as follows: The peptide length range was set to 7-52; Enzyme cutting site was trypsin/P; The maximum missed cleavage site was 2; Carbamidomethylation of cysteines as fixed modification, and oxidation of methionines and protein N-terminal acetylation as variable modifications; Protein FDR ≤ 0.01, Peptide FDR ≤ 0.01, Peptide Confidence ≥ 99%, XIC width ≤ 75 ppm. The protein quantification method was MaxLFQ. P-values and Fold change for the proteins between the two groups were calculated using the t.test function in the stats package of R. The thresholds of fold change (> 1.2 or < 0.83) and P-value < 0.05 were used to identify differentially expressed proteins. Functional annotation of all identified proteins was performed using the KEGG pathway. The microbial taxonomy/abundance was analysed based on the NCBI, and KEGG databases. Finally, the protein structures of extracellular hydrolases were subsequently constructed using SWISS-MODEL (a fully automated protein structure homology-modeling server, <https://swissmodel.expasy.org/>) based on sequences identified by metaproteomics.(3) The proteomics data of MCDF was submitted to the ProteomeXchange Consortium through the iProX partner repository with the data set ID of PXD071625.

The metabolic pathways of degrading fungal polysaccharides in the enriched MCDF-3 were identified by metagenomic analysis via the Novaseq 6000 platform (Majorbio, China). The low-quality reads (length<50 bp or with a quality value <20) were removed by fastp (version 0.23.0). Metagenomics data were assembled using MEGAHIT (version 1.1.2). The annotation of Kyoto Encyclopedia of Genes and Genomes (KEGG) was conducted using Diamond software (version 0.8.35) with an e-value of 1e-5. (2) The annotation of carbohydrate-active enzymes was conducted based on the Carbohydrate-active enzymes database with an e-value of 1e-5. (2) Gene abundance for identified enzymes was assessed using the reads per kilobase of transcript per million mapped reads method. This bacterial data of metagenomic sequencing was deposited with the BioProject accession of PRJNA1369787 in the NCBI database.

**S1.2 Native fungal diversity of waste activated sludge**


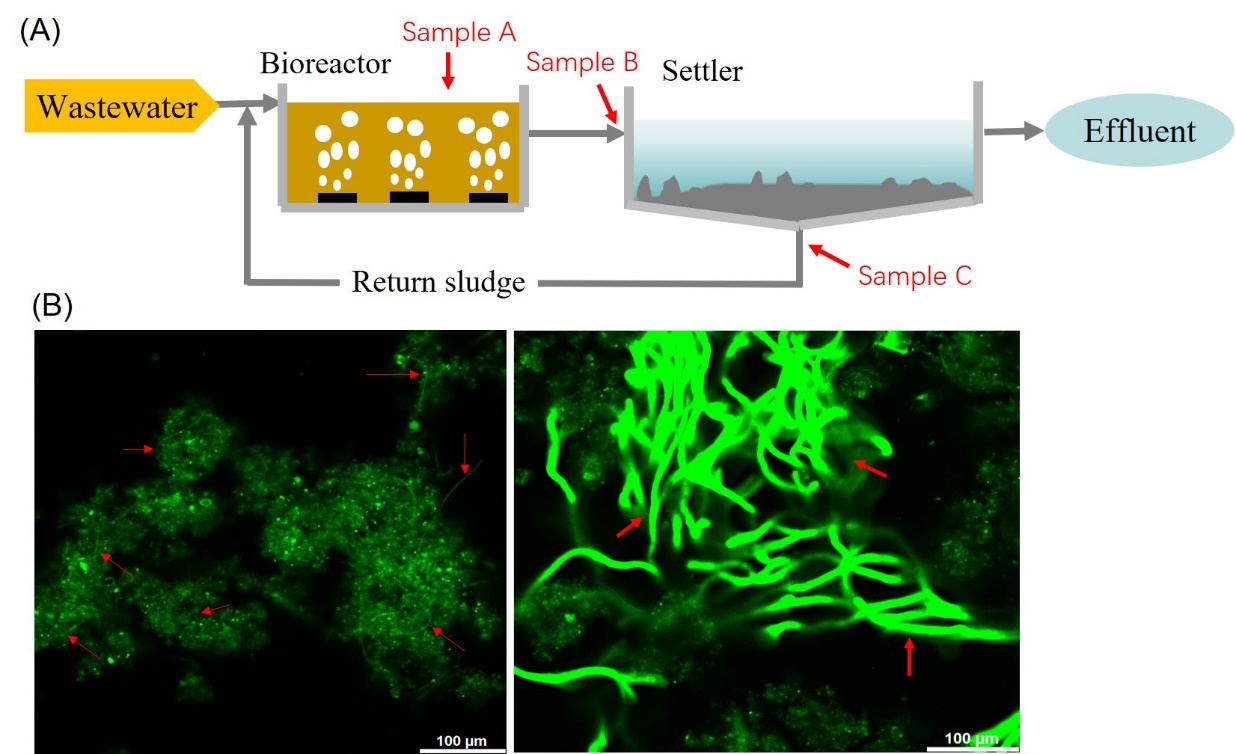


Figure S1 (A) Sampling points in a local WWTP: sample A, the bioreactor; sample B the influent of settler, and sample C, the collected WAS; (B) Calcofluor white staining of WAS

Table S2 Indices of fungi in WAS by the Illumina high-throughput sequencing

| Sample  Name | Sequence  number | Mean length (bp) | sobs | Ace | Chao | Coverage |
| --- | --- | --- | --- | --- | --- | --- |
| WAS | 115154 | 217.6 | 439.0 | 618.1 | 612.6 | 0.9701 |


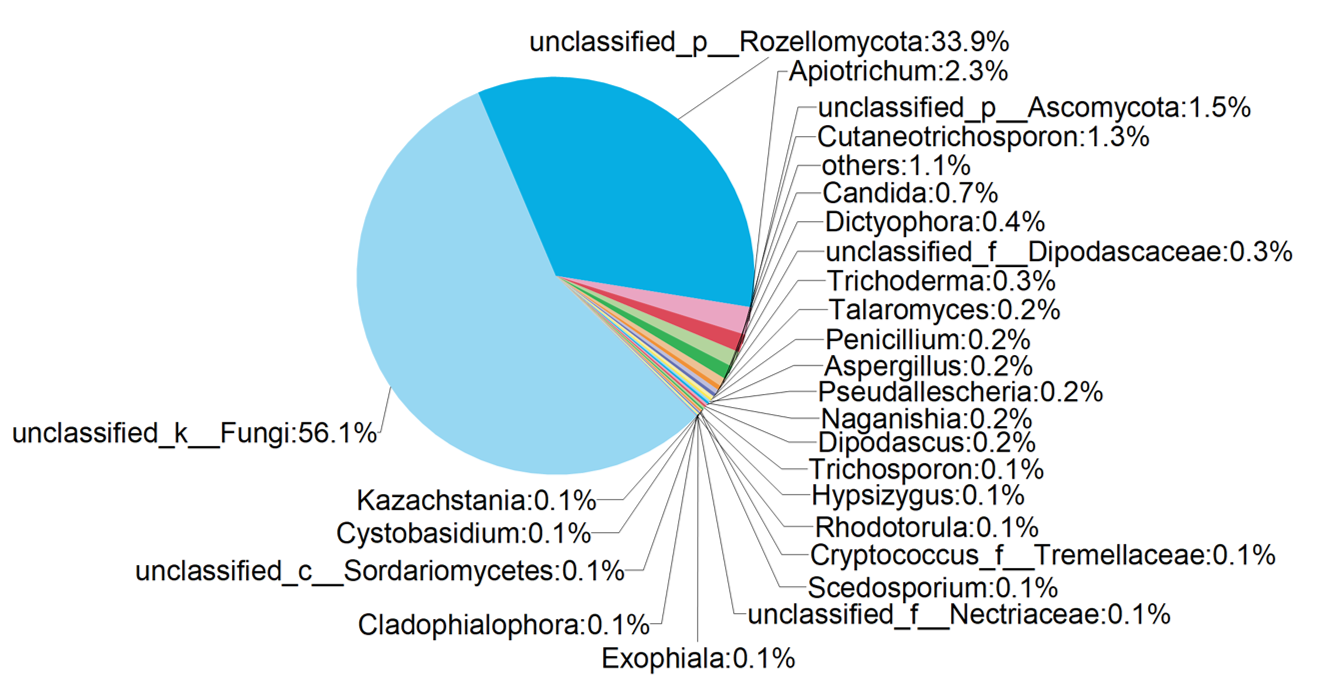


Figure S2 Fungal diversity in WAS at the genus level

**S2 WAS digestion by enriched MCDF**


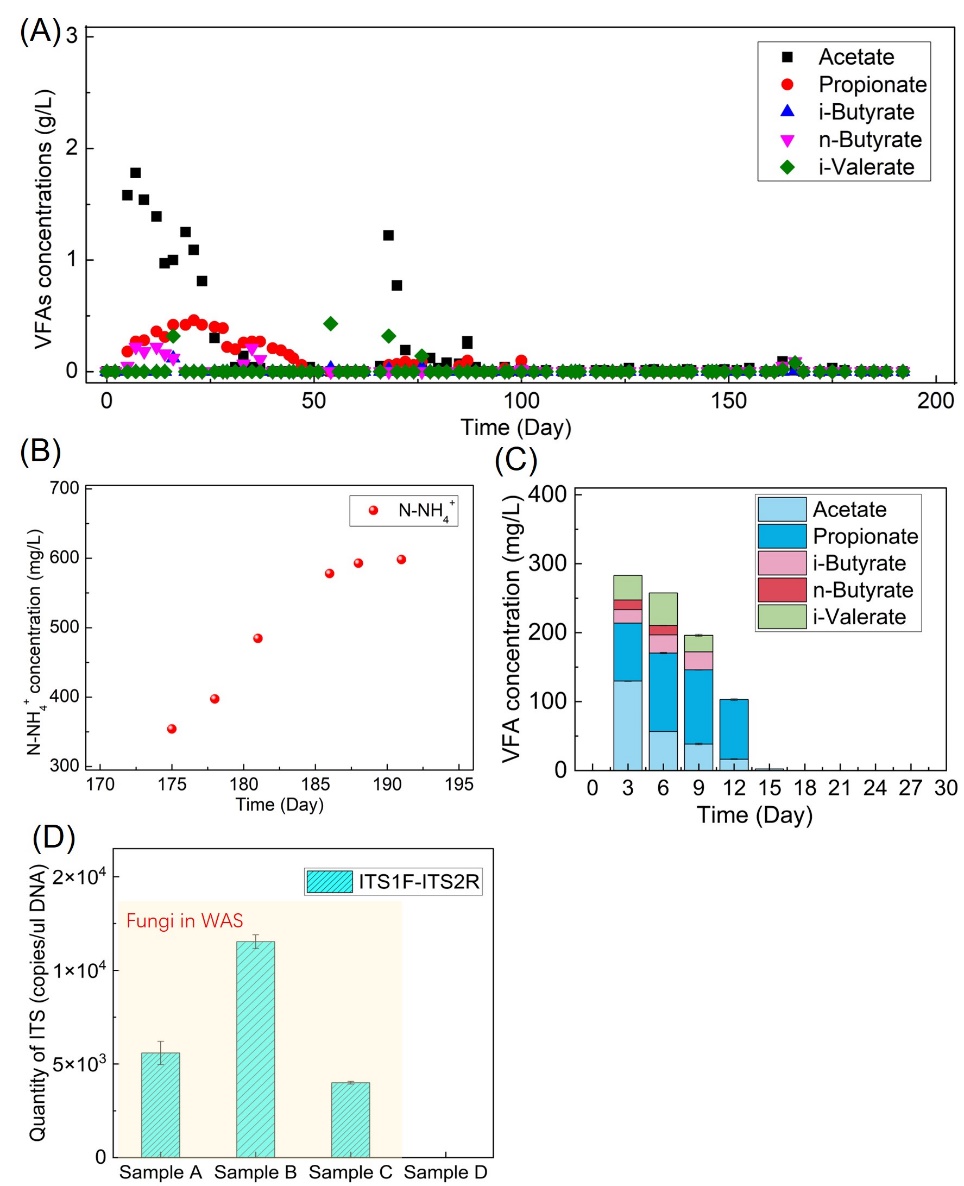


Figure S3 (A) VFAs accumulation during the MCDF enrichment, (B) N-NH_4_^+^ accumulation in the last cycle (days 174-192), (C) VFAs accumulation in WAS digestion, (D) qPCR of fungi after the WAS digestion by dosing with enriched MCDF

Table S3 Changes in particle size before and after the WAS digestion

|  | WAS-0 | WAS-30 | WAS+MCDF-30 |
| --- | --- | --- | --- |
| D10 | 23.71 ± 0.24 μm | 21.67 ± 0.14 μm | 19.62 ± 0.12 μm |
| D50 | 87.84 ± 0.45 μm | 68.4 ± 0.56 μm | 63.29 ± 0.38 μm |
| D90 | 229.1 ± 1.25 μm | 182.5 ± 2.13 μm | 166.9 ± 1.68 μm |

**S3 Anaerobic digestion of four fungi by the enriched MCDF**


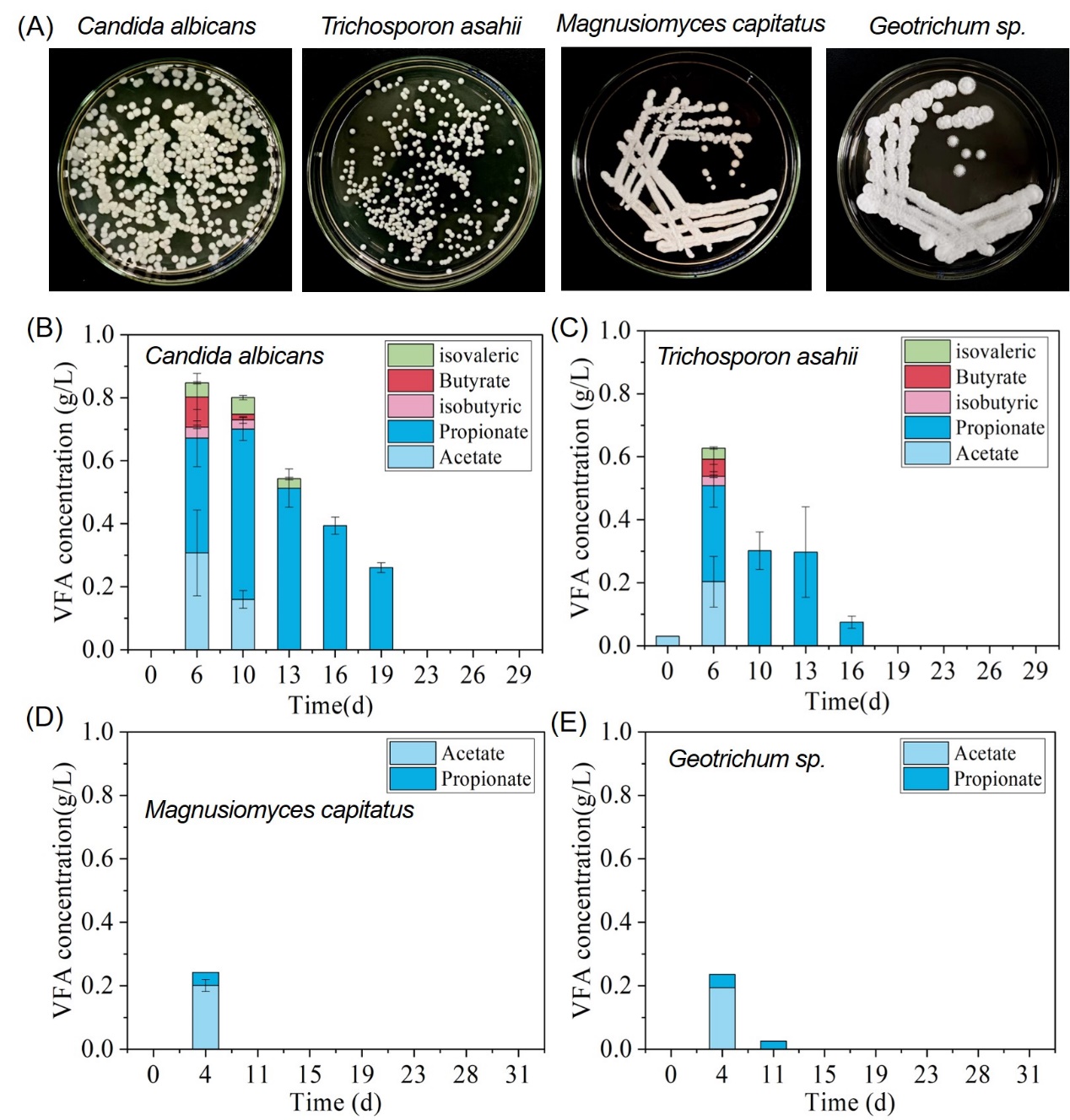


Figure S4 (A) the plaque of four fungi, and VFAs accumulation from (B) *Candida albicans*, (C) *Trichosporon asahii*, (D) *Magnusiomyces capitatus*, and (E) *Geotrichum sp.* by the enriched MCDF

**S4 Conversion of fungal components and the identification of enzymatic activities**


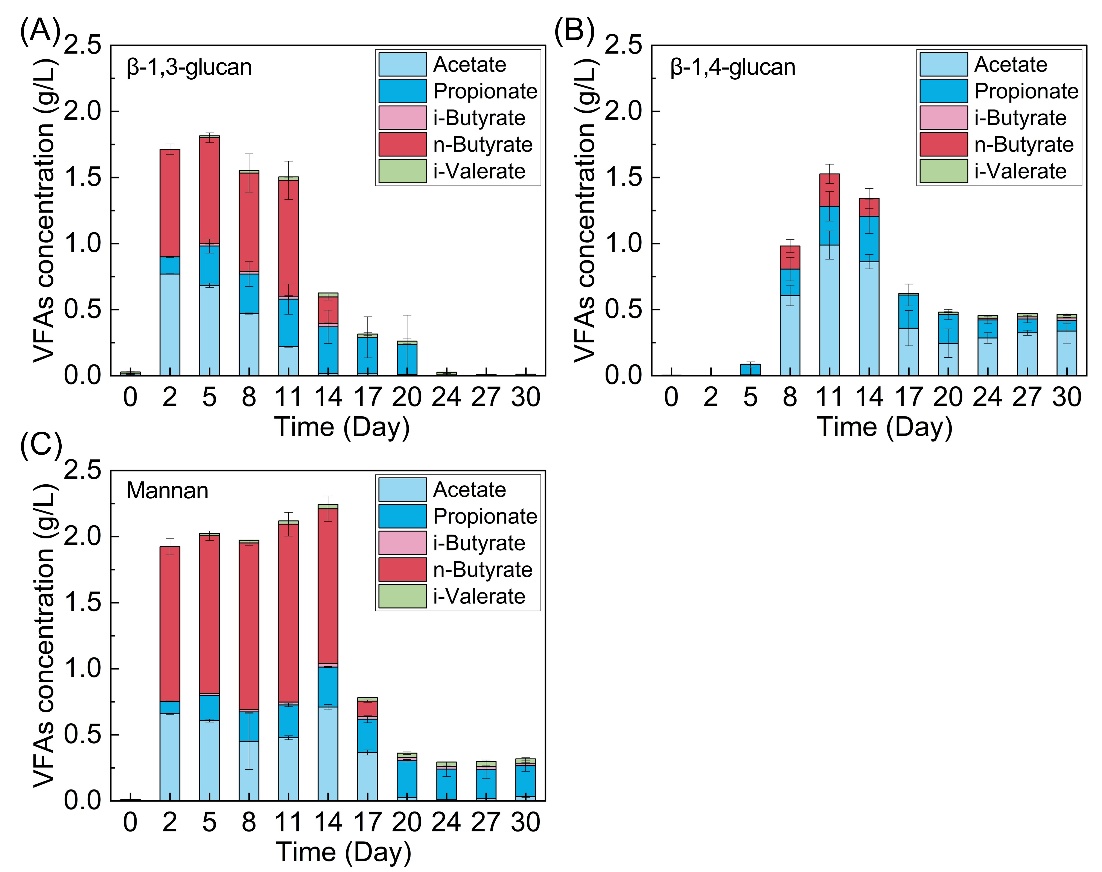


Figure S5 VFAs accumulation from (A) β-1,3-glucan, (B) β-1,4-glucan, and (C) mannan by the enriched MCDF

Table S4 Blasting results of five identified peptides with model enzymes

| Protein Accessions | Identified peptide  name | Q-Begin | Q-End | Identity  (%) | e-value | Description* |
| --- | --- | --- | --- | --- | --- | --- |
| MCDF_k97_71210_1_1 | LQNPNGYAGFR | 1 | 149 | 70.7% | 4e-70 | Chitinase (EC 3.2.1.14) of *Thermoflavimicrobium daqui* (WP_113659089.1) |
| MCDF_k97_18863_1_1 | YGYFDNENR | 50 | 113 | 96.9% | 3e-36 | Chitinodisase (EC 3.2.1.52) of *Clostridia bacterium* (MCX7746808.1) |
| MCDF_k97_112905_37_1 | IMVGADGPENR | 20 | 420 | 100% | 0 | Mannase (EC 3.2.1.78) of *Bacteroidales bacterium* (MDD3100119.1) |
| MCDF_k97_67082_40_1 | AAVIDPGADEPR | 1 | 209 | 56.8% | 3e-78 | β-1,3-glucanse (EC 3.2.1.6) of *Clostridiales bacterium* (MCR4925578.1) |
| MCDF_k97_51468_1_1 | TMLGDWDR | 6 | 193 | 76.2% | 4e-102 | β-1,4-glucanse (EC 3.2.1.4) of *Clostridiales bacterium* (MCX7749279.1) |

Note: *, the information in this section was collected from NCBI (Standard Protein BLAST)


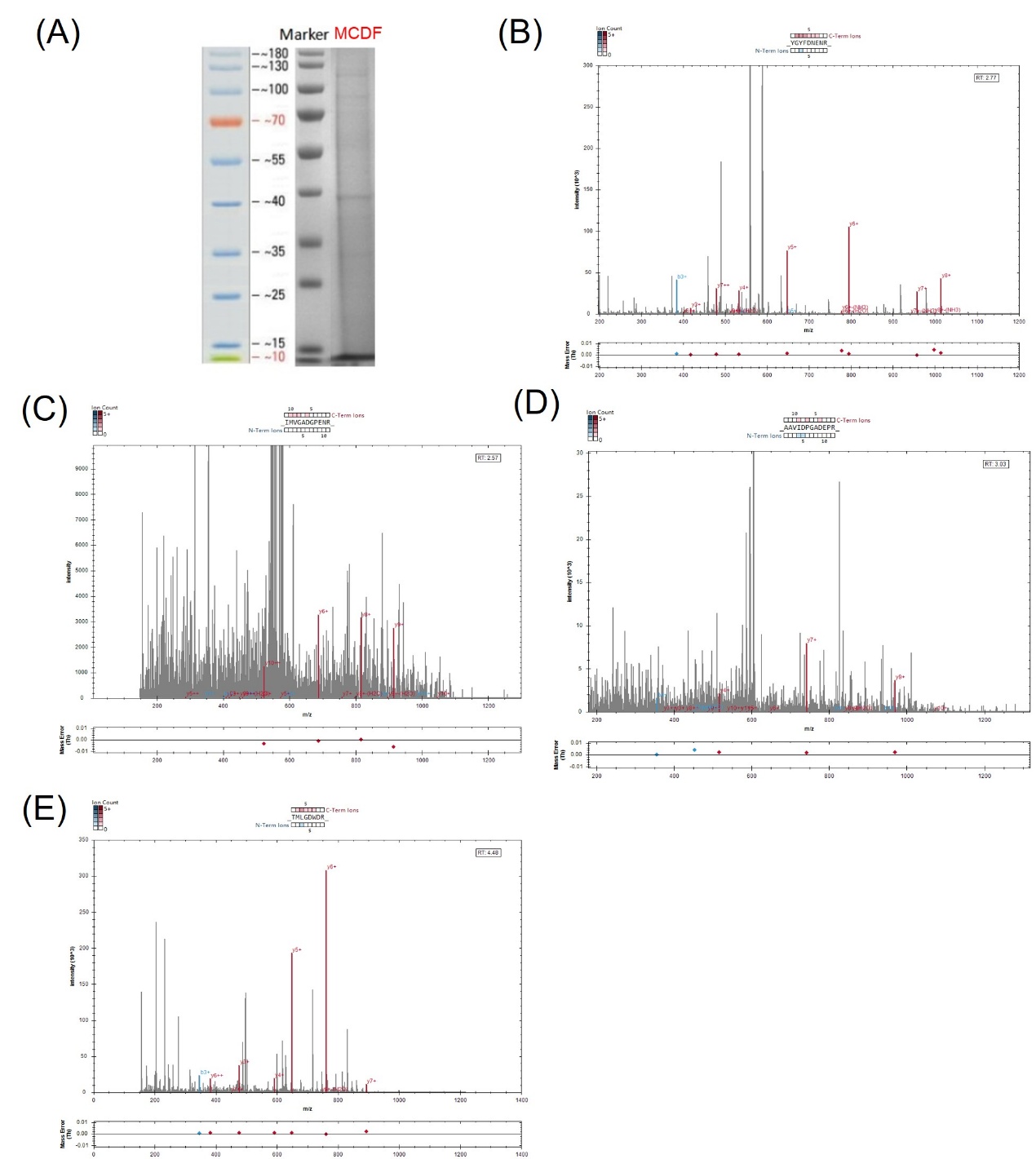


Figure S6 (A) SDS-PAGE bands of extracellular enzymes from enriched MCDF, MS2 spectrum of identified peptide in (B) Chitinodisase (EC 3.2.1.52), (C) Mannase (EC 3.2.1.78), β-1,3-glucanse (EC 3.2.1.6), and β-1,4-glucanse (EC 3.2.1.4)

**S5 Diversity of enriched MCDF by Illumina Miseq high-throughput sequencing**

Besides converges of these five samples were all above 0.999, other sequencing indices (Table S7) and curves of OTU number and Shannon index (Figure S7) all showed that the sequencing number met the demand to analyze the diversity of entire consortia.

**Table S5** Indices of enriched MCDF by the Illumina high-throughput sequencing

| Sample  name | Sequence  number | Mean length (bp) | sobs | Ace | Chao | Coverage |
| --- | --- | --- | --- | --- | --- | --- |
| WAS | 73,445 | 417.8 | 2004 | 2090.2 | 2049.1 | 0.997 |
| MCDF-1 | 44,483 | 415.2 | 197 | 224.9 | 215.6 | 0.999 |
| MCDF-2 | 43,193 | 416.6 | 221 | 263.9 | 272.0 | 0.999 |
| MCDF-3 | 52,596 | 419.4 | 191 | 222.4 | 213.9 | 0.999 |


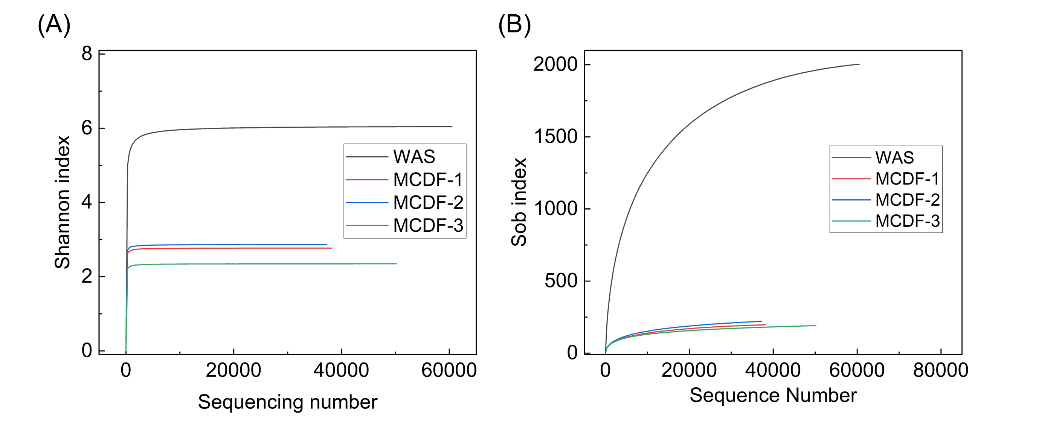


Figure S7 (A) Shannon index and (B) Sob index of enriched MCDF

**S6 Metabolic pathway of enriched MCDF by metagenomic analysis**


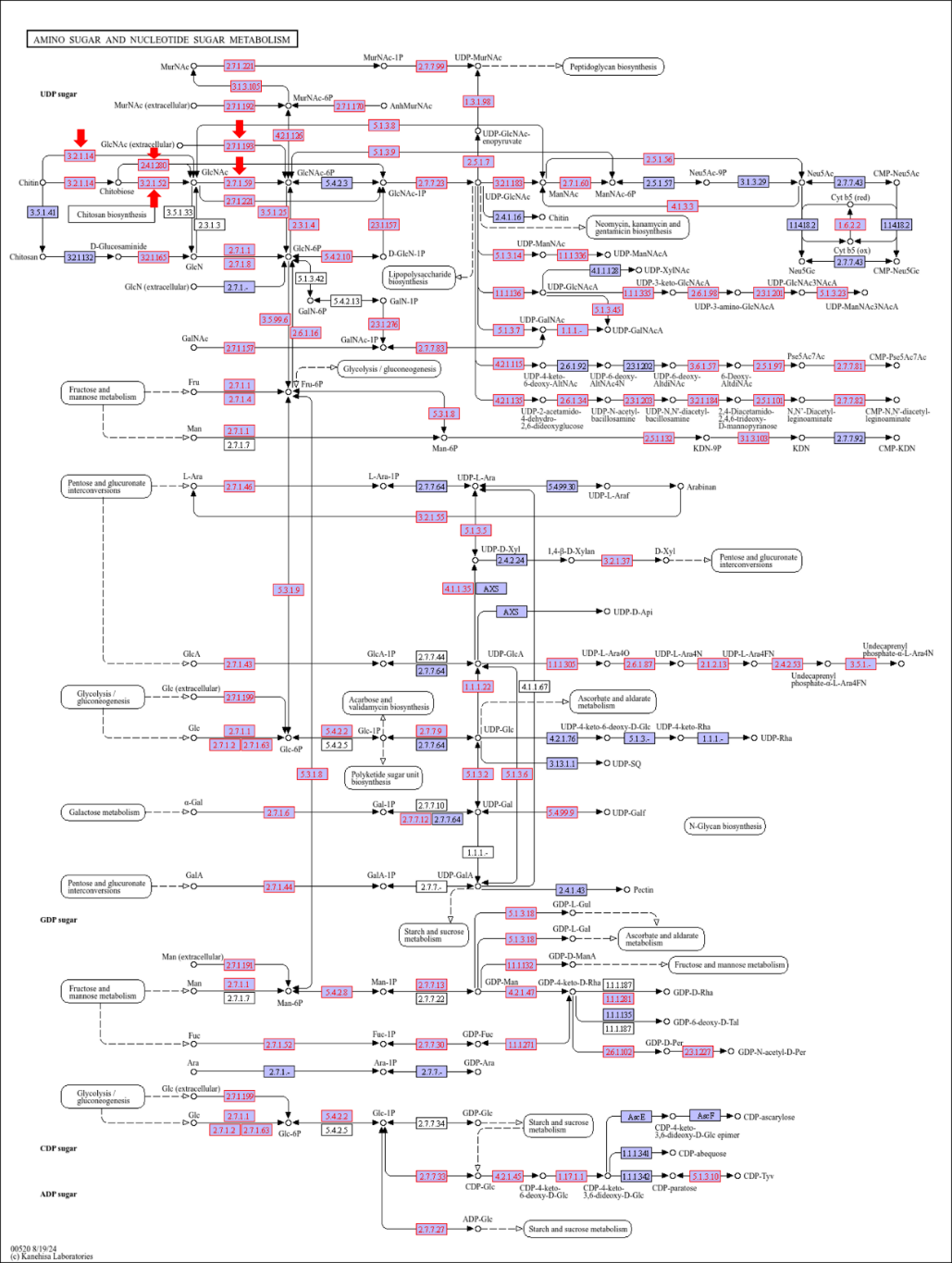


Figure S8 Identified chitinase (EC 3.2.1.14), N,N'-diacetylchitobiose phosphorylase (EC 2.4.1.280), beta-N-acetylhexosaminidase (EC 3.2.1.52), N-acetylglucosamine PTS system EIIB component (EC 2.7.1.193) and N-acetylglucosamine kinase (EC 2.7.1.59) in the enriched MCDF


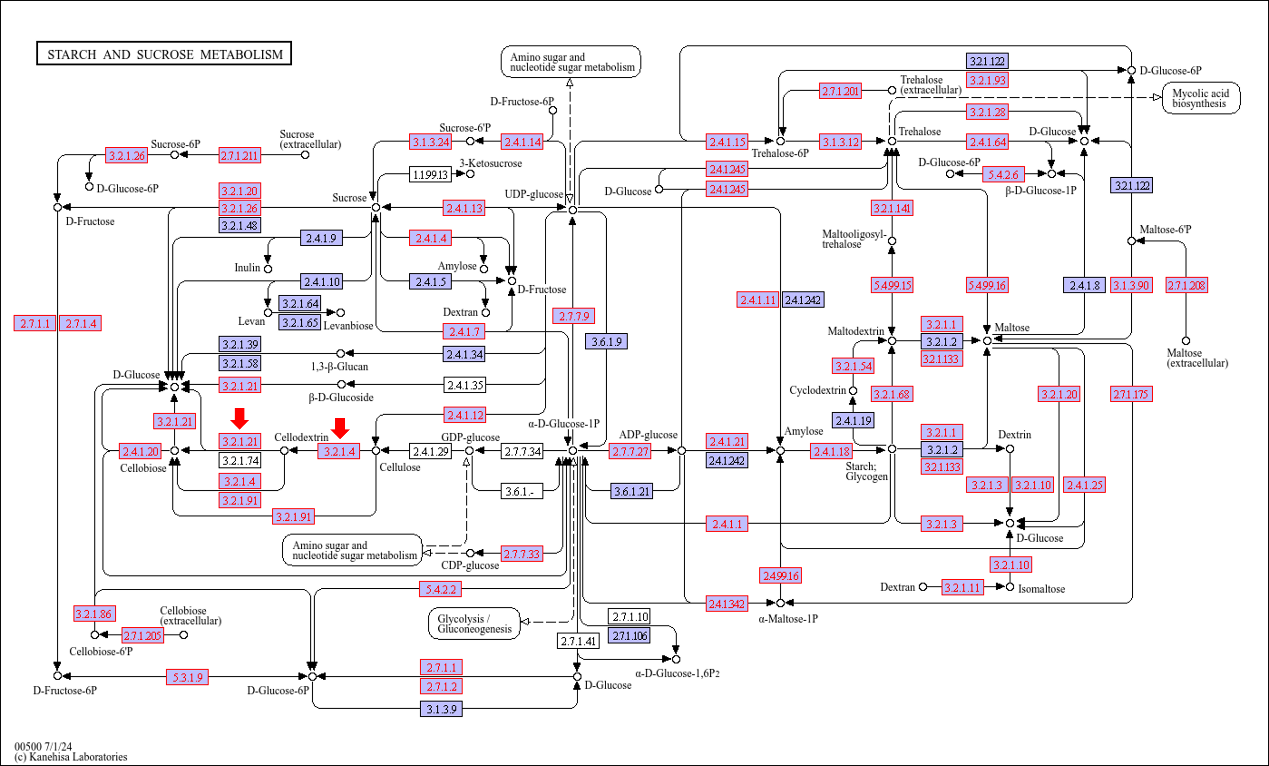


Figure S9 Identified endoglucanase (EC 3.2.1.4) and beta-glucosidase (EC 3.2.1.21) in the enriched MCDF


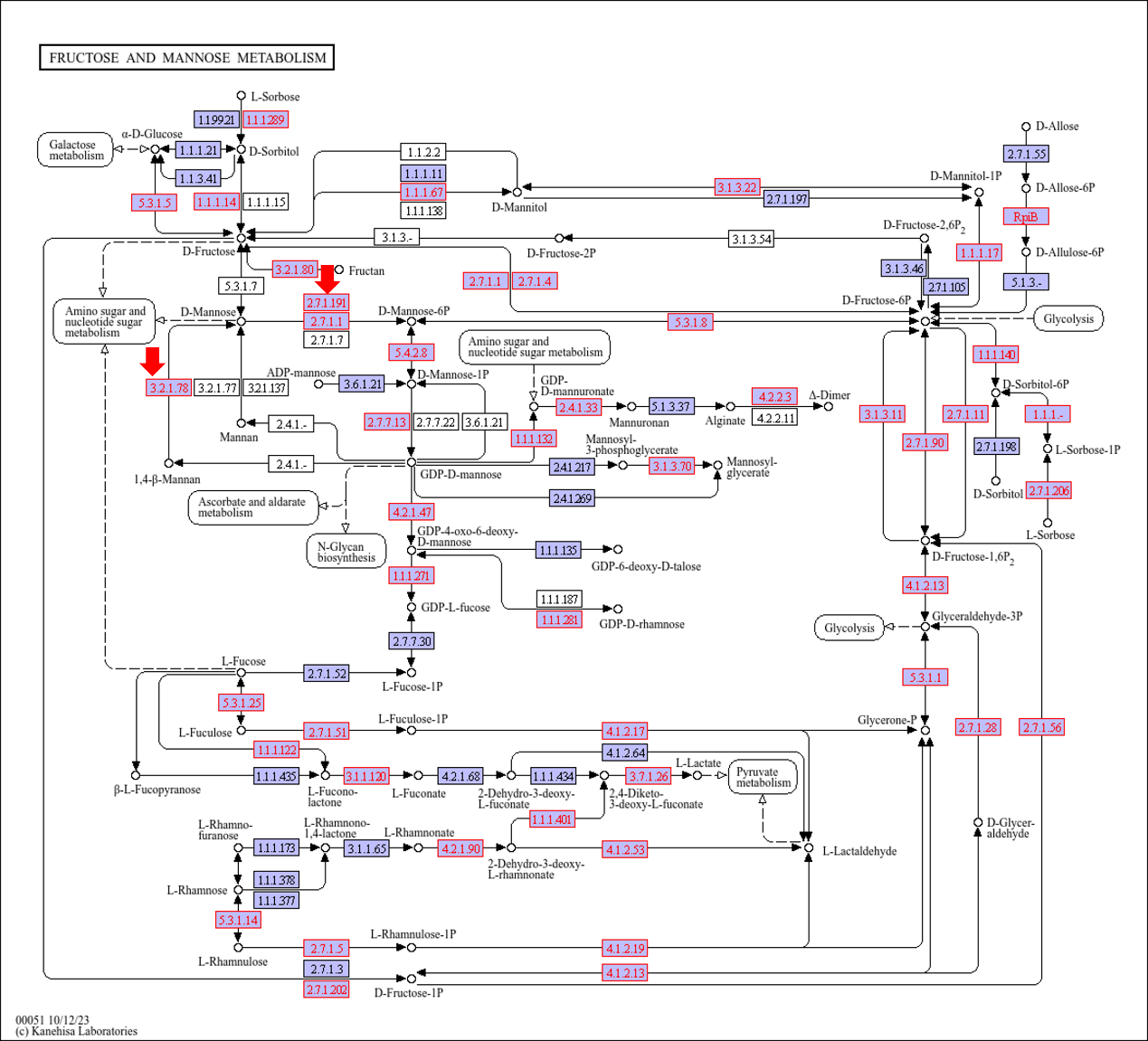


Figure S10 Identified mannan endo-1,4-beta-mannosidase (EC 3.2.1.78), mannose PTS system EIIA component (EC 2.7.1.191) and hexokinase (EC 2.7.1.1)


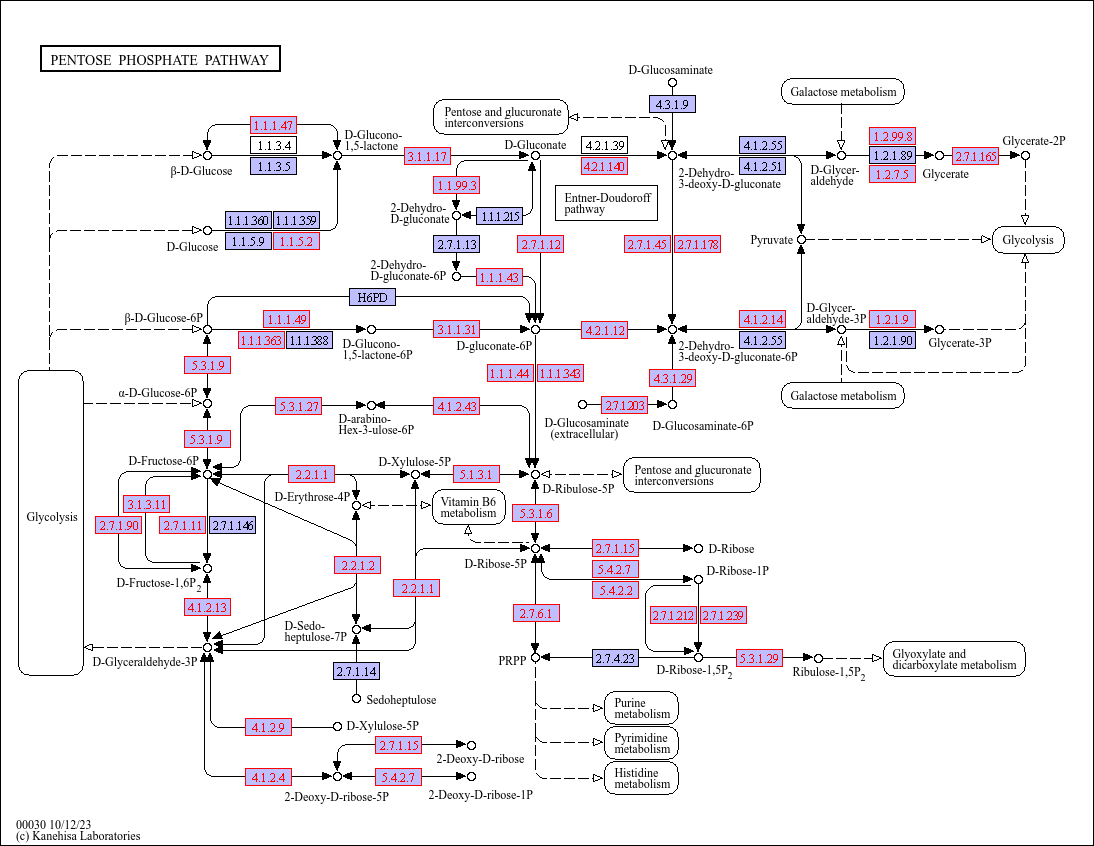


Figure S11 Identified enzymes in the PP pathway


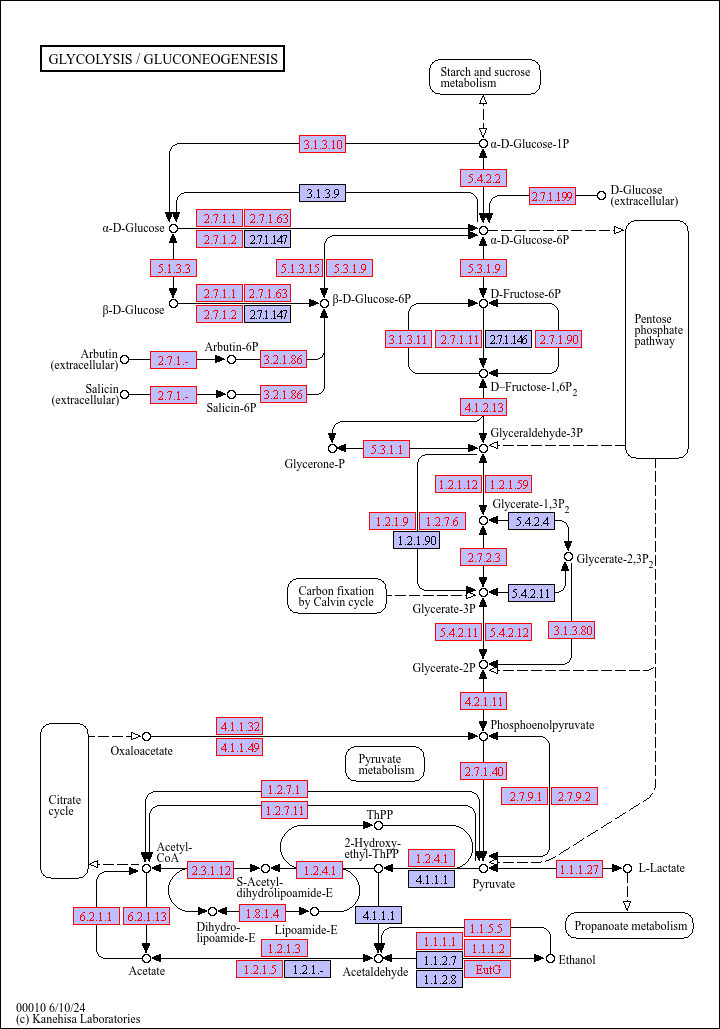


Figure S12 Identified enzymes in the EMP pathway

Table S6 Bacteria containing the chitinase (EC 3.2.1.14) by the metagenomic analysis

|  | Phylum | Genus | Species | Percentage |
| --- | --- | --- | --- | --- |
| 1 | Bacteroidota | *Proteiniphilum* | Proteiniphilum_sp. | 8.2% |
| 2 | Bacteroidota | *Petrimonas* | Petrimonas_sp. | 4.8% |
| 3 | Bacillota | *Clostridium* | Clostridium_beijerinckii | 0.16% |
|  |  |  | Clostridium_botulinum |  |
|  |  |  | Clostridium_zeae |  |
|  |  |  | Clostridium_sp._'White_wine_YQ' |  |
|  |  |  | Clostridium_beijerinckii |  |
|  |  |  | Clostridium_paraputrificum |  |
|  |  |  | Clostridium_weizhouense |  |
|  |  |  | Clostridium_cavendishii |  |
|  |  |  | Clostridium_saccharobutylicum |  |
|  |  |  | Clostridium_manihotivorum |  |
| 4 | Thermodesulfobacteriota | *Syntrophorhabdus* | Syntrophorhabdus_sp. | 0.13% |
| 5 | Bacillota | *Lachnoclostridium* | Lachnoclostridium_phytofermentans | <0.001% |
| 6 | Bacteroidota | *Alistipes* | Alistipes_sp. | <0.001% |
| 7 | Bacillota | *Anaerocolumna* | Anaerocolumna_xylanovorans | <0.001% |
|  |  |  | Anaerocolumna_aminovalerica |  |
|  |  |  | Anaerocolumna_sp. |  |
|  |  |  | Anaerocolumna_jejuensis |  |
| 8 | Bacteroidota | *Bacteroides* | Bacteroides_intestinalis | <0.001% |
| 9 | Pseudomonadota | *Candidatus_Accumulibacter* | Candidatus_Accumulibacter_sp. | <0.001% |
| 10 | Bacillota | *Enterococcus* | Enterococcus_villorum | <0.001% |
| 11 | Chloroflexota | *Herpetosiphon* | Herpetosiphon_sp. | <0.001% |
| 12 | Bacteroidota | *Macellibacteroides* | Macellibacteroides_sp._HH-ZS | <0.001% |
| 13 | Bacillota | *Paenibacillus* | Paenibacillus_frigoriresistens | <0.001% |
|  |  |  | Paenibacillus_alvei | <0.001% |
|  |  |  | Paenibacillus_tepidiphilus | <0.001% |
|  |  |  | Paenibacillus_sp._MER_TA_81-3 | <0.001% |
|  |  |  | Paenibacillus_sp._FPU-7 | <0.001% |
|  |  |  | Paenibacillus_elgii | <0.001% |
| 14 | Bacillota | *Ruminiclostridium* | Ruminiclostridium_cellobioparum | <0.001% |
|  |  |  | Ruminiclostridium_sufflavum |  |
| 15 | Bacillota | *Ruminococcus* | Ruminococcus_sp. | <0.001% |
| 16 | Bacillota | *Staphylococcus* | Staphylococcus_aureus | <0.001% |

Table S7 Bacteria containing the beta-N-acetylhexosaminidase (EC 3.2.1.52) by the metagenomic analysis

|  | Phylum | Genus | Species | Percentage |
| --- | --- | --- | --- | --- |
| 1 | Bacteroidota | *Fermentimonas* | Fermentimonas_caenicola | 42.0% |
|  |  |  | Fermentimonas_sp. |  |
| 2 | Thermotogota | *Mesotoga* | Mesotoga_sp._H07pep.5.4 | 13.8% |
|  |  |  | Mesotoga_sp. |  |
| 3 | Bacteroidota | *Proteiniphilum* | Proteiniphilum_saccharofermentans | 8.2% |
|  |  |  | Proteiniphilum_acetatigenes |  |
|  |  |  | Proteiniphilum_sp._X52 |  |
|  |  |  | Proteiniphilum_sp._UBA5346 |  |
|  |  |  | Proteiniphilum_sp._UBA5510 |  |
|  |  |  | Proteiniphilum_propionicum |  |
|  |  |  | Proteiniphilum_sp._UBA5480 |  |
|  |  |  | Proteiniphilum_sp._UBA5431 |  |
|  |  |  | Proteiniphilum_sp. |  |
| 4 | Bacteroidota | *Petrimonas* | Petrimonas_sp._IBARAKI | 4.8% |
|  |  |  | Petrimonas_sp. |  |
| 5 | Thermodesulfobacteriota | *Syntrophobacter* | Syntrophobacter_fumaroxidans | 0.61% |
| 6 | Chloroflexota | *Ornatilinea* | Ornatilinea_apprima | 0.53% |
| 7 | Spirochaetota | *Sphaerochaeta* | Sphaerochaeta_sp._UBA5836 | 0.27% |
|  |  |  | Sphaerochaeta_sp. |  |
| 8 | Bacillota | *Clostridium* | Clostridium_sp._CAG:678 | 0.16% |
| 9 | Bacteroidota | *Lentimicrobium* | Lentimicrobium_saccharophilum | 0.10% |
|  |  |  | Lentimicrobium_sp. |  |
| 10 | Bacillota | *Syntrophomonas* | Syntrophomonas_sp. | 0.05% |
| 11 | Bacteroidota | *Mariniphaga* | Mariniphaga_anaerophila | 0.04% |
| 12 | Bacillota | *Lachnoclostridium* | Lachnoclostridium_sp. | 0.01% |
| 13 | Spirochaetota | *Treponema* | Treponema_sp. | 0.004% |
| 14 | Pseudomonadota | *Undibacterium* | Undibacterium_sp. | <0.001% |
| 15 | Pseudomonadota | *Stenotrophomonas* | Stenotrophomonas_acidaminiphila | <0.001% |
| 16 | Pseudomonadota | *Rhodanobacter* | Rhodanobacter_sp._C03 | <0.001% |
| 17 | Bacteroidota | *Prevotella* | Prevotella_sp._CAG:924 | <0.001% |
| 18 | Bacteroidota | *Phaeodactylibacter* | Phaeodactylibacter_sp. | <0.001% |
| 19 | Bacteroidota | *Parabacteroides* | Parabacteroides_faecis | <0.001% |
|  |  |  | Parabacteroides_sp._AM08-6 |  |
| 20 | Ignavibacteriota | *Ignavibacterium* | Ignavibacterium_sp. | <0.001% |
|  |  |  | Ignavibacterium_album |  |
| 21 | Bacteroidota | *Dysgonomonas* | Dysgonomonas_capnocytophagoides | <0.001% |
| 22 | Pseudomonadota | *Devosia* | Devosia_sp. | <0.001% |
| 23 | Thermodesulfobacteriota | *Desulforhabdus* | Desulforhabdus_amnigena | <0.001% |
| 24 | Deinococcota | *Deinococcus* | Deinococcus_sp. | <0.001% |
| 25 | Acidobacteriota | *Bryobacter* | Bryobacter_sp. | <0.001% |
| 26 | Bacteroidota | *Bacteroides* | Bacteroides_oleiciplenus | <0.001% |
| 27 | Armatimonadota | *Armatimonas* | Armatimonas_rosea | <0.001% |
| 28 | Chloroflexota | *Anaerolinea* | Anaerolinea_sp. | <0.001% |
| 29 | Bacteroidota | *Alistipes* | Alistipes_sp. | <0.001% |
| 30 | Bacillota | *Acetivibrio* | Acetivibrio_mesophilus | <0.001% |
| 31 | Bacillota | *Paenibacillus* | Paenibacillus_whitsoniae | <0.001% |
|  |  |  | Paenibacillus_koleovorans |  |
|  |  |  | Paenibacillus_herberti |  |
|  |  |  | Paenibacillus_apiarius |  |
|  |  |  | Paenibacillus_oleatilyticus |  |
|  |  |  | Paenibacillus_marchantiophytorum |  |
|  |  |  | Paenibacillus_elgii |  |

Table S8 Bacteria containing the mannan endo-1,4-beta-mannosidase (EC 3.2.1.78) by the metagenomic analysis

|  | Phylum | Genus | Species | Percentage |
| --- | --- | --- | --- | --- |
| 1 | Bacteroidota | *Fermentimonas* | Fermentimonas_caenicola | 42.0% |
| 2 | Bacteroidota | *Petrimonas* | Petrimonas_sp. | 4.8% |
| 3 | Bacillota | *Syntrophomonas* | Syntrophomonas_sp. | 0.05% |
| 4 | Thermodesulfobacteriota | *Desulforhabdus* | Desulforhabdus_amnigena | <0.001% |
| 5 | Bacillota | *Paenibacillus* | Paenibacillus_athensensis | <0.001% |
| 6 | Bacteroidota | *Parabacteroides* | Parabacteroides_chartae | <0.001% |
| 7 | Bacteroidota | *Bacteroides* | Bacteroides_luti | <0.001% |
|  |  |  | Bacteroides_sp. |  |

Table S9 Bacteria containing the endo-1,3(4)-beta-glucanase (EC 3.2.1.6) by the metagenomic analysis

|  | Phylum | Genus | Species | Percentage |
| --- | --- | --- | --- | --- |
| 1 | Thermodesulfobacteriota | *Syntrophorhabdus* | Syntrophorhabdus_sp. | 0.13% |

Table S10 Bacteria containing the endo-glucanase (EC 3.2.1.4) by the metagenomic analysis

|  | Phylum | Genus | Species | Percentage |
| --- | --- | --- | --- | --- |
| 1 | Thermotogota | *Mesotoga* | *Mesotoga_sp.* | 13.78% |
| 2 | Bacteroidota | *Proteiniphilum* | *Proteiniphilum_sp._UBA5346* | 8.17% |
|  |  |  | *Proteiniphilum_sp._X52* |  |
|  |  |  | *Proteiniphilum_saccharofermentans* |  |
|  |  |  | *Proteiniphilum_propionicum* |  |
|  |  |  | *Proteiniphilum_sp.* |  |
|  |  |  | *Proteiniphilum_acetatigenes* |  |
|  |  |  | *Proteiniphilum_sp._X52* |  |
|  |  |  | *Proteiniphilum_acetatigenes* |  |
| 3 | Bacteroidota | *Petrimonas* | *Petrimonas_sp.* | 4.75% |
| 4 | Candidatus_Cloacimonadota | *Candidatus_Cloacimonas* | *Candidatus_Cloacimonas_sp.* | 0.22% |
| 5 | Bacillota | *Clostridium* | *Clostridium_sp.* | 0.16% |
| 6 | Bacteroidota | *Lentimicrobium* | *Lentimicrobium_saccharophilum* | 0.10% |
| 7 | Bacteroidota | *Mariniphaga* | *Mariniphaga_sp.* | 0.04% |
| 8 | Spirochaetota | *Treponema* | *Treponema_sp._CETP13* | 0.004% |
| 9 | Bacillota | *Anaerocolumna* | Anaerocolumna_aminovalerica | <0.001% |
| 10 | Chloroflexota | *Anaerolinea* | Anaerolinea_sp. | <0.001% |
|  |  |  | Anaerolinea_thermolimosa |  |
| 11 | Bacteroidota | *Bacteroides* | Bacteroides_sp. | <0.001% |
| 12 | Bacteroidota | *Dysgonomonas* | Dysgonomonas_sp._HDW5A | <0.001% |
| 13 | Ignavibacteriota | *Ignavibacterium* | Ignavibacterium_album | <0.001% |
|  |  |  | Ignavibacterium_sp. |  |
| 14 | Bacillota | *Paenibacillus* | Paenibacillus_terricola | <0.001% |
| 15 | Bacteroidota | *Paludibacter* | Paludibacter_sp. | <0.001% |
| 16 | Bacteroidota | *Parabacteroides* | Parabacteroides_sp._Marseille-P3160 | <0.001% |
|  |  |  | Parabacteroides_timonensis |  |
| 17 | Bacillota | *Ruminococcus* | Ruminococcus_sp. | <0.001% |
| 18 | Spirochaetota | *Spirochaeta* | Spirochaeta_lutea | <0.001% |
| 19 | Candidatus_Moranbacteria | *unclassified_Candidatus_Moranbacteria* | Candidatus_Moranbacteria_bacterium | <0.001% |

**References:**

1. APHA; AWWA; WEF, *Standard methods for the examination of water and wastewater*. 2005.

2. Ren, Y.; Yu, G.; Shi, C.; Liu, L.; Guo, Q.; Han, C.; Zhang, D.; Zhang, L.; Liu, B.; Gao, H.; Zeng, J.; Zhou, Y.; Qiu, Y.; Wei, J.; Luo, Y.; Zhu, F.; Li, X.; Wu, Q.; Li, B.; Fu, W.; Tong, Y.; Meng, J.; Fang, Y.; Dong, J.; Feng, Y.; Xie, S.; Yang, Q.; Yang, H.; Wang, Y.; Zhang, J.; Gu, H.; Xuan, H.; Zou, G.; Luo, C.; Huang, L.; Yang, B.; Dong, Y.; Zhao, J.; Han, J.; Zhang, X.; Huang, H., Majorbio Cloud: A one-stop, comprehensive bioinformatic platform for multiomics analyses. *iMeta* **2022,** *1*, (2), e12.

3. Waterhouse, A.; Bertoni, M.; Bienert, S.; Studer, G.; Tauriello, G.; Gumienny, R.; Heer, F. T.; de Beer, T. A P.; Rempfer, C.; Bordoli, L.; Lepore, R.; Schwede, T., SWISS-MODEL: homology modelling of protein structures and complexes. *Nucleic Acids Research* **2018,** *46*, (W1), W296-W303.
